# Mutational signatures reveal the role of RAD52 in p53-independent p21-driven genomic instability

**DOI:** 10.1186/s13059-018-1401-9

**Published:** 2018-03-16

**Authors:** Panagiotis Galanos, George Pappas, Alexander Polyzos, Athanassios Kotsinas, Ioanna Svolaki, Nickolaos N. Giakoumakis, Christina Glytsou, Ioannis S. Pateras, Umakanta Swain, Vassilis L. Souliotis, Alexandros G. Georgakilas, Nicholas Geacintov, Luca Scorrano, Claudia Lukas, Jiri Lukas, Zvi Livneh, Zoi Lygerou, Dipanjan Chowdhury, Claus Storgaard Sørensen, Jiri Bartek, Vassilis G. Gorgoulis

**Affiliations:** 1 Molecular Carcinogenesis Group, Department of Histology and Embryology, School of Medicine, National Kapodistrian University of Athens, 75 Mikras Asias Str, GR-11527 Athens, Greece; 2 Danish Cancer Society Research Centre, Strandboulevarden 49, DK-2100 Copenhagen, Denmark; 3 Biomedical Research Foundation of the Academy of Athens, 4 Soranou Ephessiou Str, GR-11527 Athens, Greece; 4 Laboratory of Biology, School of Medicine, University of Patras, 26505 Patras, Rio Greece; 5 Department of Biology, University of Padova, 35121 Padova, Italy; 6 Department of Biomolecular Sciences, Weizmann Institute of Science, 76100 Rehovot, Israel; 7 Institute of Biology, Medicinal Chemistry and Biotechnology, National Hellenic Research Foundation, 48 Vassileos Constantinou Ave, GR-11635 Athens, Greece; 8 Physics Department, School of Applied Mathematical and Physical Sciences, National Technical University of Athens (NTUA), 15780 Zografou, Athens Greece; 9 Department of Chemistry, New York University, New York, 10012 USA; 10 Novo Nordisk Foundation Center for Protein Research, Faculty of Health and Medical Sciences, University of Copenhagen, Copenhagen, Denmark; 11 Department of Radiation Oncology, Dana-Farber Cancer Institute, 450 Brookline Ave, Boston, MA 02215 USA; 12 Harvard Medical School, 25 Shattuck St, Boston, MA 02115 USA; 13 Biotech Research and Innovation Centre (BRIC), University of Copenhagen, Ole Maaloes Vej 5, DK-2200 Copenhagen, Denmark; 14 Science for Life Laboratory, Division of Genome Biology, Department of Medical Biochemistry and Biophysics, Karolinska Institute, SE-171 77 Stockholm, Sweden; 15 Faculty of Biology, Medicine and Health, University of Manchester, Manchester Academic Health Science Centre, Wilmslow Road, Manchester, M20 4QL UK

**Keywords:** p21^WAF1/Cip1^, Rad52, Genomic instability, Translesion DNA synthesis (TLS), Single nucleotide substitution (SNS), Break-induced replication (BIR), Single strand annealing (SSA)

## Abstract

**Background:**

Genomic instability promotes evolution and heterogeneity of tumors. Unraveling its mechanistic basis is essential for the design of appropriate therapeutic strategies. In a previous study, we reported an unexpected oncogenic property of p21^WAF1/Cip1^, showing that its chronic expression in a p53-deficient environment causes genomic instability by deregulation of the replication licensing machinery.

**Results:**

We now demonstrate that p21^WAF1/Cip1^ can further fuel genomic instability by suppressing the repair capacity of low- and high-fidelity pathways that deal with nucleotide abnormalities. Consequently, fewer single nucleotide substitutions (SNSs) occur, while formation of highly deleterious DNA double-strand breaks (DSBs) is enhanced, crafting a characteristic mutational signature landscape. Guided by the mutational signatures formed, we find that the DSBs are repaired by Rad52-dependent break-induced replication (BIR) and single-strand annealing (SSA) repair pathways. Conversely, the error-free synthesis-dependent strand annealing (SDSA) repair route is deficient. Surprisingly, Rad52 is activated transcriptionally in an E2F1-dependent manner, rather than post-translationally as is common for DNA repair factor activation.

**Conclusions:**

Our results signify the importance of mutational signatures as guides to disclose the repair history leading to genomic instability. We unveil how chronic p21^WAF1/Cip1^ expression rewires the repair process and identifies Rad52 as a source of genomic instability and a candidate therapeutic target.

**Electronic supplementary material:**

The online version of this article (10.1186/s13059-018-1401-9) contains supplementary material, which is available to authorized users.

## Background

Genomic instability is a hallmark of cancer that plays an important role in shaping tumor behavior over time [1–5]. Elucidating the molecular routes that drive this phenomenon is essential for designing proper therapeutic strategies as well as monitoring the natural history of carcinogenesis [6]. Not all genetic alterations are *driver* events, since each type of cancer bears a large number of *passenger* mutations [7]. Although the latter have no causative role in cancer development, they represent pieces of a *mutational signature* pattern that can provide information about the type(s) of DNA damage taking place and the repair pathway(s) involved [7, 8].

We have recently reported that precancerous- and cancerous-associated chronic p21^WAF1/Cip1^ expression, in a p53-deficient environment, fuels genomic instability by deregulating the replication licensing machinery, causing re-replication, a deleterious form of replication stress. These events occur throughout a senescence-like phase during which an error-prone DNA repair process takes place, forming a genetic landscape that allows a subpopulation of p21^WAF1/Cip1^–expressing cells to escape senescence (termed “escaped” cells) [9]. Interestingly, and in accordance with the oncogene-induced DNA damage model for cancer development [4], the escaped cells demonstrated aggressive features and increased chemo-resistance [9].

Which particular error-prone repair pathway(s) is(are) employed by the p21^WAF1/Cip1^-expressing cells to craft the permissive environment for “senescence escape” is a key question, as its answer would unveil potential genomic instability routes that could represent future therapeutic targets. To address this question we followed a reverse engineering approach examining the *mutational signature* patterns of the escaped cells (Additional file 1: Figure S1). The *mutational signatures* that include amount and type of single nucleotide substitutions (SNSs), insertions and deletions (INDELs), and breakpoint junctions reflect the actual “repair history” that takes place following exogenous and/or endogenous mutagenic events. As a guideline we utilized the 21 distinct *mutational signatures* reported by Alexandrov and collegues, who extracted them after analyzing ~ 5 × 10^6^ mutations from ~ 7000 cancers [8]. We demonstrate that p53-independent elevated p21^WAF1/Cip1^ expression drives genomic instability by rewiring the global cellular DNA repair landscape towards predominantly error-prone processes that prominently rely on the RAD52 recombinase.

## Results

### The SNS load is reduced in p21^WAF1/Cip1^ escaped cells

**Fig. 1:**
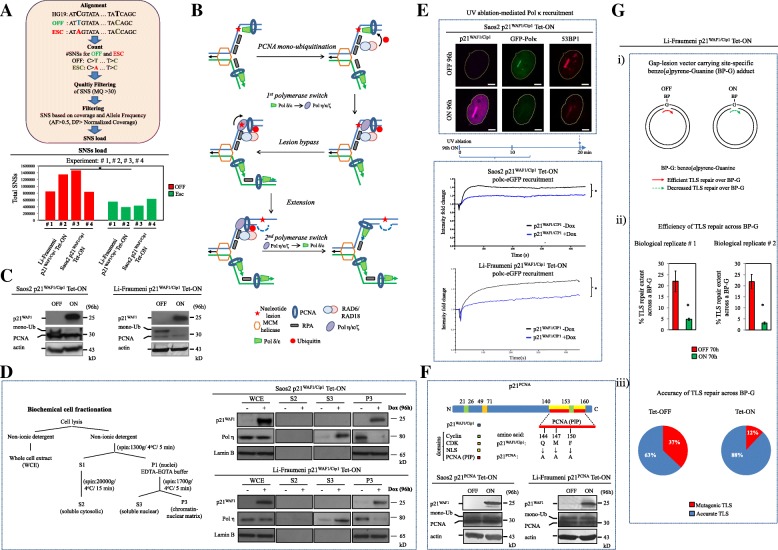
Reduction of single nucleotide substitution (SNS) and malfunction of the translesion DNA synthesis and repair (TLS) process upon protracted p21^WAF1/Cip1^ expression. **a** Chronic p21^WAF1/Cip1^ expression, in a p53-deficient environment, leads to the emergence of a subpopulation of p21^WAF1/Cip1^ aggressive and chemo-resistant (escaped (*ESC*)) cells, after bypassing an initial senescence-like phase, that carry a lower SNS “load” [9]. SNS identification and filtering were performed with the use of Samtools and VCFtools in non-induced and escaped (Saos2- and Li-Fraumeni-p21^WAF1/Cip1^ Tet-ON) cells (see also Additional file 1: Figure S1), depicted in accompanying histograms (*p < 0.05 (Saos2 and Li-Fraumeni), OFF vs ESC, Welch’s *t*-test) (for details see “Methods” section). **b** TLS pathway function. TLS is a DNA damage tolerance process enabling the DNA replication machinery to replicate over DNA lesions. Upon DNA damage PCNA is mono-ubiquitinated, followed by polymerase switch from normal high-fidelity DNA replication polymerases to TLS ones. TLS polymerase Polη, bound to PCNA, inserts a nucleotide opposite to the lesion and, assisted or not by an additional TLS polymerase like Polκ or Polζ, extends beyond the insertion. Finally, a second polymerase switch takes place by substituting TLS polymerases with high-fidelity ones. **c** Sustained p21^WAF1/Cip1^ expression results in decreased mono-ubiquitination of PCNA (*mono-Ub*). Immunoblots (IBs) in 96-h induced Saos2- and Li-Fraumeni-p21^WAF1/Cip1^ Tet-ON cells (*n* = 3 experiments). **d** Reduced binding of Polη to chromatin in cells with protracted p21^WAF1/Cip1^ expression. IBs after cell fractionation (described in scheme) depicting lower levels of Polη in chromatin extracts from 96-h induced Saos2- and Li-Fraumeni-p21^WAF1/Cip1^ Tet-ON cells (*n* = 3 experiments). **e** Immunofluorescent confocal microscopy (*top panel*) showing reduced Polκ loading on regions of damaged chromatin after UV-laser ablation in 96-h induced Saos2-p21^WAF1/Cip1^ Tet-ON cells transfected with a GFP-Polκ vector. Plots (*lower panel*) depict recruitment kinetics of Polκ in Saos2- and Li-Fraumeni- p21^WAF1/Cip1^ Tet-ON cells, respectively (see also Additional files 2, 3, 4 and 5). The average intensity of fluorescence at the site of damage and the total cell fluorescence with respect to time were quantified and plotted. Five cells in each condition of three independent experiments were processed. Time frames for obtaining IFs and recruitment plots are depicted in *middle panel*. **f** A specific p21^WAF1/Cip1^ mutant (p21^PCNA^; harboring Q144, M147, F150 substitutions to A in its PCNA-interacting-protein (PIP) degron motif) with an abrogated interaction with PCNA [9]. IBs depict mono-ubiquitination of PCNA (*mono-Ub*) in 96-h induced Saos2- and Li-Fraumeni-p21^PCNA^ Tet-ON cells (*n* = 3 experiments). **g** Overexpression of p21^WAF1/Cip1^ decreases TLS repair efficiency across a site-specific lesion in a gapped plasmid TLS assay (*i*). Induced Li-Fraumeni-p21^WAF1/Cip1^ Tet-ON cells were assayed for TLS efficiency (*ii*) and accuracy of repair (*iii*) with a gap-lesion vector carrying a site-specific benzo[*a*]pyrene-guanine (*BP-G*) adduct (*i*) (Additional file 6: Table S1) (*n* = 3 experiments). Actin and lamin B serve as loading control (* p < 0.05, error bars indicate SDs). MQ mapping quality, AF allele frequency, DP sequencing depth, MCM Mini-Chromosome Maintenance protein complex, RPA Replication Protein A, WCE whole cell extract, S2 soluble cytosolic, S3 soluble nuclear, P3 chromatin-nuclear matrix

As a first step we evaluated the SNS load in the human p21^WAF1/Cip1^ escaped cell models [9], following the experimental algorithm described in Additional file 1: Figure S1. We found that they harbor fewer SNSs compared to the p21^WAF1/Cip1^-un-induced controls (Fig. 1a). This result was cell type independent, consistently observed in both p21^WAF1/Cip1^-inducible cellular systems examined, namely cancerous Saos2 cells and non-cancerous cells from a Li-Fraumeni syndrome patient (Fig. 1a). Given that protracted p21^WAF1/Cip1^ expression in a p53-null environment promotes genomic instability [9], the reduced number of SNSs in the escaped cells seemed at first glance counterintuitive. Yet, from a broader perspective, even though SNSs represent genetic defects, they signify the “last option” the cell possesses to avoid the more detrimental double strand break (DSB) lesions [7]. Translesion DNA synthesis (TLS) or DNA damage tolerance (DDT) is the main cellular repair mode that orchestrates the choice of the above mentioned last option by dealing with all types of SNSs that remain unrepaired by the cellular high-fidelity repair mechanisms (Additional file 1: Figure S2b). TLS is also known as post-replication repair (PRR) as it initiates DNA synthesis downstream of a DNA lesion, thus allowing repair after DNA synthesis (Fig. 1b) [10, 11]. Hence, the reduced amount of SNSs in the escaped cells could result from a malfunctioning TLS/DDT repair process and/or reduced activity of one or more high-fidelity repair pathways that commonly mend defective nucleotides (Additional file 1: Figure S2c).

### Reduced SNS load reflects malfunctioning TLS and nucleotide excision repair (NER) and base excision repair (BER) pathways

A key step regulating TLS is monoubiquitination of PCNA (Ub-PCNA), at Lys 164 (K164), carried out by the E3-ligase Rad18 [12]. Such monoubiquitinated PCNA (Mono-Ub-PCNA) operates as a “molecular switch”, shifting normal DNA replication into TLS. Mono-Ub-PCNA increases its affinity for TLS polymerase Polη, a Y-family polymerase that inserts a nucleotide directly opposite a nucleotide lesion (Fig. 1b) [13]. Although Polη is involved in error-free lesion bypass of UV-induced thymidine (TT) dimers [14, 15], it can also catalyze error-prone bypass of other lesion types such as 8-oxoguanine, apurinic/apyrimidinic (AP) sites, and DNA adducts caused by benzo[*a*]pyrene diol epoxide (BPDE), leading to mutagenic consequences [16]. Following p21^WAF1/Cip1^ induction, both the mono-Ub-PCNA and the chromatin bound fraction of Polη were dramatically reduced, consistent with our conjecture that dysfunctional TLS may be responsible for the reduced SNS load in the escaped cells (Fig. 1c, d). In line with the above, recruitment of Polκ, an extender in translesion synthesis [17], to sites of UV-laser induced DNA damage was significantly decreased in cells expressing p21^WAF1/Cip1^ (Fig. 1e; Additional files 2, 3, 4, and 5). Supporting the notion that the induced p21^WAF1/Cip1^ binds to and inhibits PCNA monoubiquitination, and consequently reduces recruitment of the TLS polymerases [18], replacing the wild-type p21^WAF1/Cip1^ by a p21 mutant defective in PCNA binding (p21^PCNA^) did not reduce PCNA monoubiquitination (Fig. 1f). To assess whether the above biochemical traits reflect dysfunctional TLS repair we employed a gapped plasmid TLS assay [19] to quantify the extent of repair across a site-specific (benzo[*a*]pyrene-guanine) adduct and found that induction of p21^WAF1/Cip1^ resulted in a robustly decreased (4.6–7.1-fold) frequency and concomitantly lower repair accuracy (12 instead of 37 %) (Fig. 1g; Additional file 6: Table S1).


**Additional file 2:** Video time-laps imaging showing Polκ recruitment at sites of DNA damage after UV induced laser ablation in non-induced Saos2-p21^WAF1/Cip1^ (Tet-OFF) cells. (AVI 20511 kb)



**Additional file 3:** Video time-laps imaging showing Polκ recruitment at sites of DNA damage after UV induced laser ablation in induced Saos2-p21^WAF1/Cip1^ Cip1 (Tet-ON) cells. (AVI 2159 kb)



**Additional file 4:** Video time-laps imaging showing Polκ recruitment at sites of DNA damage after UV induced laser ablation in non-induced Li-Fraumeni-p21^WAF1/Cip1^ (Tet-OFF) cells. (AVI 23548 kb)



**Additional file 5:** Video time-laps imaging showing Polκ recruitment at sites of DNA damage after UV induced laser ablation in induced Li-Fraumeni-p21^WAF1/Cip1^ (Tet-ON) cells. (AVI 50041 kb)



**Additional file 7:** Video time-laps imaging showing Rad52 recruitment at sites of DNA damage after UV induced laser ablation in non-induced Saos2-p21^WAF1/Cip1^ (Tet-OFF) cells. (AVI 44036 kb)



**Additional file 8:** Video time-laps imaging showing Rad52 recruitment at sites of DNA damage after UV induced laser ablation in induced Saos2-p21^WAF1/Cip1^ (Tet-ON) cells. (AVI 3557 kb)



**Additional file 9:** Video time-laps imaging showing Rad52 recruitment at sites of DNA damage after UV induced laser ablation in non-induced Li-Fraumeni-p21 ^WAF1/Cip1^ (Tet-OFF) cells. (AVI 1628 kb)



**Additional file 10:** Video time-laps imaging showing Rad52 recruitment at sites of DNA damage after UV induced laser ablation in induced Li-Fraumeni-p21^WAF1/Cip^ (Tet-ON) cells. (AVI 5150 kb)


**Fig. 2:**
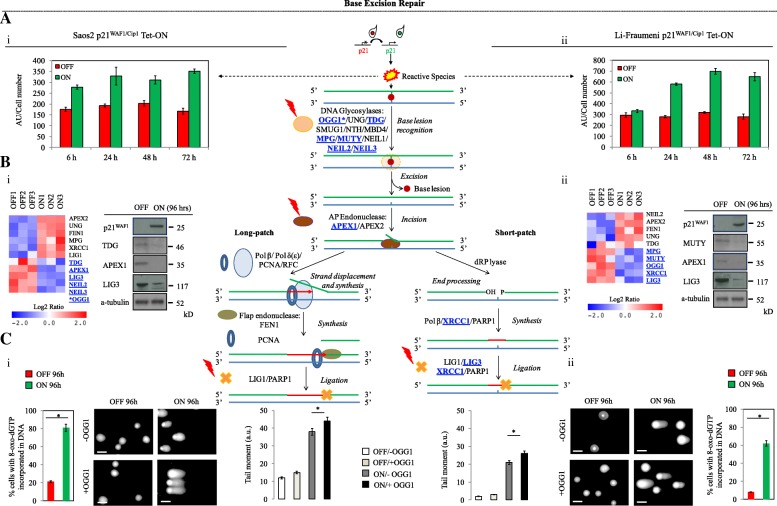
Decreased activity of the base excision repair (BER) pathway in cells with sustained p21^WAF1/Cip1^ expression. **a** Increased reactive species (RS) levels were assessed with a DCFH-DA assay in Saos2 (*i*) and Li-Fraumeni (*ii*) cells with protracted p21^WAF1/Cip1^ expression (*p < 0.05 (Saos2), *p = 0.05 (Li-Fraumeni), *t*-test; error bars indicate standard deviation; *n* = 5 experiments). As shown in the middle panel RS production can lead to generation of base/nucleotide oxidative lesions. **b** RNAseq analysis showed that essential factors of the BER pathway were statistically significantly down-regulated (*p* ≤ 0.05) in 96-h induced Saos2- (*i*) and Li-Fraumeni- (*ii*) p21^WAF1/Cip1^ Tet-ON cells (see also Additional file 1: Figure S3 for specific real time RT-PCR validation). Note that although in Saos2- p21^WAF1/Cip1^ Tet-ON cells OGG1 expression was not found by RNAseq analysis, specific real-time RT-PCR and microarray analysis (see also Additional file 1: Figure S3) [9] confirmed its decreased expression. Selective immunoblots for APEX1, LIG3, TDG, and MUTY confirmed the specificity of the RNA analysis results. Note that LIG3 participates also in mismatch repair (MMR; Additional file 1: Figure S3). α-Tubulin served as loading control. **c** Modified alkaline Comet assay demonstrated the presence of oxidized purines like 8-oxo-dG in 96-h induced Saos2- (*i*) and Li-Fraumeni- (*ii*) p21^WAF1/Cip1^ Tet-ON cells, using 8-oxoguanine glycosylase (OGG1) (*p < 0.05 (Saos2), *p = 0.05 (Li-Fraumeni), *t*-test; error bars indicate standard deviation; *n* = 5 experiments). Comet data were corroborated by an 8-oxo-dG-specific assay measuring DNA incorporation of 8-oxo-dG in p21^WAF1/Cip1^-expressing cells [24], which indicated lower OGG1 activity (*p < 0.05 (Saos2), *p = 0.05 (Li-Fraumeni), *t*-test; error bars indicate standard deviation; *n* = 5 experiments). Consequently, as depicted in the model in the *middle panel*, recognition and excision of the affected nucleotide lesion is impaired in the BER process. The *middle panel* depicts the components and steps during BER. The BER pathway is responsible for removal of small lesions from DNA, especially oxidized, alkylated, deaminated bases and abasic sites. BER can be induced by oxidative stress and various genotoxic insults. Its specificity relies on the excision of base damage by glycosylases. In humans, the mechanism of BER involves the initial action of DNA glycosylases followed by the processing of the resulting abasic site either by the AP-lyase activity of the glycosylases or by the apurinic/apyrimidic endonucleases APE1/APE2, which incise the DNA strand. The resulting single-strand break can be processed by two BER subpathways. Either the short-patch branch is engaged, if a single nucleotide is replaced, or the long-patch branch, if 2–10 new nucleotides are synthesized. *OGG1* 8-oxoguanine DNA glycosylase, *UNG* uracil DNA glycosylase, *TDG* thymine DNA glycosylase, *SMUG1* single-strand-selective monofunctional uracil-DNA glycosylase 1, *NTH* DNA glycosylase and apyrimidinic (AP) lyase (endonuclease III), *MBD4* methyl-CpG binding domain 4, DNA glycosylase, *MPG* N-methylpurine DNA glycosylase, *MUTY* adenine DNA glycosylase, *NEIL1/2/3* Nei-like DNA glycosylase 1/ 2/ 3, *APEX1/2* apurinic/apyrimidinic endodeoxyribonuclease 1/2, *POLB*/*POLD*, DNA polymerase beta/ delta, *PCNA* proliferating cell nuclear antigen, *RFC* replication factor C, *FEN1* flap structure-specific endonuclease 1, *LIG1*/*LIG3* DNA ligase 1/3, *PARP1* poly(ADP-ribose) polymerase 1, *XRCC1* X-ray repair cross complementing 1

**Fig. 3:**
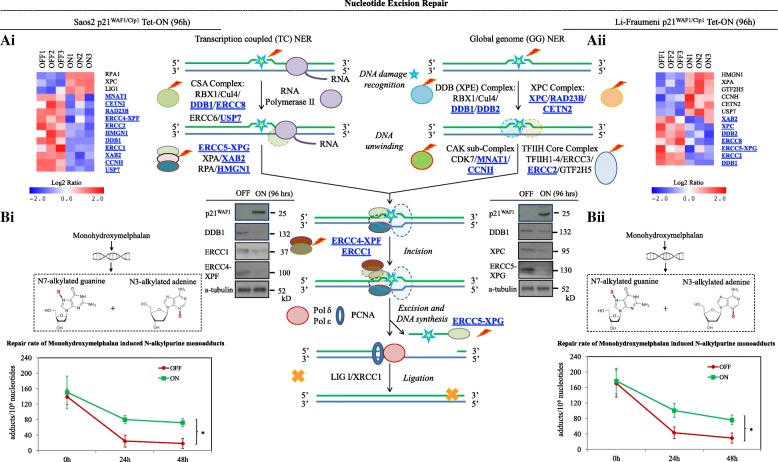
Decreased activity of the nucleotide excision repair (NER) pathway in cells with prolonged p21^WAF1/Cip1^ expression. **a** RNAseq analysis showed that essential factors of the NER pathway were statistically significantly down-regulated (*p* ≤ 0.05; see also Additional file 1: Figure S3 for specific real-time RT-PCR validation) in 96-h induced Saos2- (*i*) and Li-Fraumeni- (*ii*) p21^WAF1/Cip1^ Tet-ON cells. Selective immunoblots for DDB1, ERCC1, ERCC4-XPF, XPC, and ERCC5-XPG, key factors in NER [22], confirming the specificity of the RT-PCR results. α-Tubulin served as loading control; the same protein extracts were used as in Fig. 2. **b** Decreased repair capacity of N-alkylpurine monoadducts in induced Saos2- (*i*) and Li-Fraumeni- (*ii*) p21^WAF1/Cip1^ Tet-ON cells and treated with monohydroxymelphalan, an inducer of specific NER substrates [69–71]. The data shown are based on five independent experiments with at least two analyses for independent experiment/independent experiment experiment (* p < 0.05, error bars indicate SDs). The *middle panel* depicts the components and function of NER. The NER pathway is responsible for repair of bulky lesions, especially UV-induced thymine dimers and 6,4-photoproducts, as well as non-bulky ones. Following DNA damage recognition, a short single-stranded DNA fragment that contains the lesion is removed. The remaining undamaged single-stranded DNA segment is used by DNA polymerase as a template to synthesize the complementary sequence. Final ligation to complete NER and formation of a double-stranded DNA is carried out by DNA ligase. Depending on how the DNA damage is recognized, NER can be divided into two subpathways: transcription coupled NER (*TC-NER*) and global genome NER (*GG-NER*). While the two subpathways differ in how they recognize DNA damage, they share the same process for lesion incision, repair, and ligation. *RBX1* Ring-box 1, *Cul4* Cullin 4, *DDB1/2* Damage specific DNA binding protein 1/2, *ERCC8 (CSA)* ERCC excision repair 8, CSA ubiquitin ligase complex subunit, *ERCC6 (CSB)* ERCC excision repair 6, chromatin remodeling factor, *USP7* Ubiquitin-specific peptidase 7; *ERCC4-XPF* Excision repair 4, endonuclease, *ERCC5-XPG* ERCC excision repair 5, endonuclease, *XPA* XPA, DNA damage recognition and repair factor, *XAB2* XPA binding protein 2, *RPA* Replication protein A, *HMGN1* High mobility group nucleosome binding domain 1; *XPC* XPC complex subunit, DNA damage recognition and repair factor, *RAD23B* RAD23 homolog B, *CETN2* Centrin 2, *CDK7* Cyclin-dependent kinase 7, *MNAT1* CDK activating kinase assembly factor, *CCNH* Cyclin H, *TFIIH1–4* Transcription/repair factor IIH 1–4, *ERCC3* ERCC excision repair 3, TFIIH core complex helicase subunit, *ERCC2* ERCC excision repair 2, TFIIH core complex helicase subunit, *TTDA (GTF2H5/TFB5)* General transcription factor IIH subunit 5

An additional feature that could further exacerbate the impact of malfunctioning TLS-mediated repair would be potentially increased numbers of erroneous nucleotides in the genome due to deregulation of the high-fidelity nucleotide repair mechanisms. It has been reported that p21^WAF1/Cip1^ can negatively modulate high-fidelity DNA repair processes, particularly those implicated in excising defective nucleotides, such as nucleotide excision repair (NER), mismatch repair (MMR), and base excision repair (BER) [20]. Given that our experimental systems were not exposed to exogenous causes of DNA damage, such as UV or X-ray irradiation, a major potential source of nucleotide abnormalities could be an endogenous process, particularly over-production of reactive oxygen species (ROS) [21]. Elevated ROS would lead to nucleotide oxidative lesions, with 8-oxo-dGuanine (8-oxo-dG) being the most frequent [22]. Indeed, p21^WAF1/Cip1^ induction was followed by a progressive generation of ROS (Fig. 2a), in agreement with previous findings [23]. Applying a modified version of the alkaline Comet assay, we could detect oxidized purines (like 8-oxo-dG) in the DNA of the p21^WAF1/Cip1^-induced cells using OGG1 (8-oxoguanine glycosylase) as a damage probe (Fig. 2). Moreover, RNAseq and protein analysis showed that essential factors of the nucleotide repair mechanisms were down-regulated (Figs. 2 and 3; Additional file 1: Figures S3 and S4). BER, which is mainly responsible for removing oxidative lesions [22], was particularly affected, as the levels of several key DNA glycosylases and downstream effectors were down-regulated (Fig. 2b; Additional file 1: Figures S3 and S4). Consistently, we found enhanced DNA incorporation of 8-oxo-dG in p21^WAF1/Cip1^-expressing cells, using an 8-oxo-dG-specific assay [24], indicative of lower OGG1 activity (Fig. 2c). NER was disrupted as well, as judged from the levels of its components and the repair capacity of N-alkylpurine monoaducts [25, 26] (Fig. 3; Additional file 1: Figures S3, S4, and S5). Although NER is primarily involved in repairing bulky DNA lesions, for instance UV-induced TT dimers, it can also repair non-bulky nucleotide defects [25]. Of note, a few key BER and NER factors demonstrated a differential expression pattern (Figs. 2 and 3; Additional file 1: Figure S4). Given the redundant nature of BER glycosylases [22] (Additional file 1: Figure S4a), and that most suppressed NER factors resided in DNA damage-recognition complexes (Additional file 1: Figure S4b), we can infer this seemingly “confusing” result as a cellular context-based inefficient compensatory response to restore functionality [27–29].

Overall, due to elevated reactive oxygen species (ROS) and malfunctioning BER and NER, the amount of unrepaired oxidative lesions, such as 8-oxo-dG, would increase over time, further burdening the cells with dysfunctional TLS. Consequently, apart from re-replicated DNA [9], the unrepaired nucleotides may represent an additional source of replication fork stalling, collapse, and DNA DSBs (Additional file 1: Figure S2c).

### Protracted p21^WAF1/Cip1^ expression fosters Rad52-dependent break-induced replication (BIR) and single strand annealing (SSA)

**Fig. 4:**
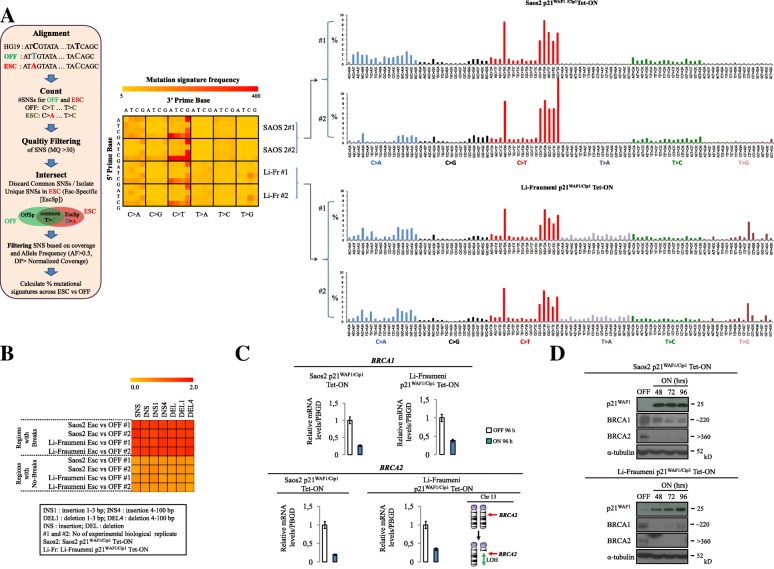
Extended p21^WAF1/Cip1^ over-expression shapes the mutational signature landscape. **a** Escaped (30 days induced) Saos2- and Li-Fraumeni-p21^WAF1/Cip1^ Tet-ON cells exhibit specific patterns of single nucleotide substitution (SNS). SNSs with mapping quality above 30 found only in the escaped cells were filtered based on sequencing depth and scored as ESC-specific (see also Additional file 1: Figure S1). Those SNSs were used to calculate the mutational signature of ESC versus OFF cells (for details see “Methods” section and Additional file 1: Figure S1). Heat map shows the number of mutation type at each mutation context, which was corrected for the frequency of each triplet in the human genome (hg19). Histograms present the mutation-type frequency at each mutation context from two biological replicates of escaped Saos2- and Li-Fraumeni-p21^WAF1/Cip1^ Tet-ON cells, respectively. Both presentations show reproducible patterns of the mutational signatures 6, 15, 3 [8]. **b** Heat map showing the association of SNSs, nucleotide insertions (*INS*), and nucleotide deletions (*DEL*) with the observed chromosomal breakpoints (±50 kb around the breakpoint) versus the remaining genome in escaped Saos2- and Li-Fraumeni-p21^WAF1/Cip1^ Tet-ON cells. **c** Real-time RT-PCR assessment of BRCA1 and BRCA2 mRNA expression in induced and non-induced Saos2 and Li-Fraumeni p21^WAF1/Cip1^ Tet-ON cells (*p < 0.05 (Saos2), *p = 0.05 (Li-Fraumeni), *t*-test; error bars indicate standard deviation; *n* = 3 experiments). Loss of heterozygosity at the q arm of chromosome 13 (which hosts the *BRCA2* locus (q13.10)) in induced Li-Fraumeni-p21^WAF1/Cip1^Tet-ON cells [9]**. d** Immunoblots depict reduced BRCA1 and BRCA2 expression in induced Saos2- and Li-Fraumeni-p21^WAF1/Cip1^ Tet-ON cells at the indicated time points. α-Tubulin served as loading control. MQ mapping quality, AF allele frequency, DP sequencing depth

**Fig. 5:**
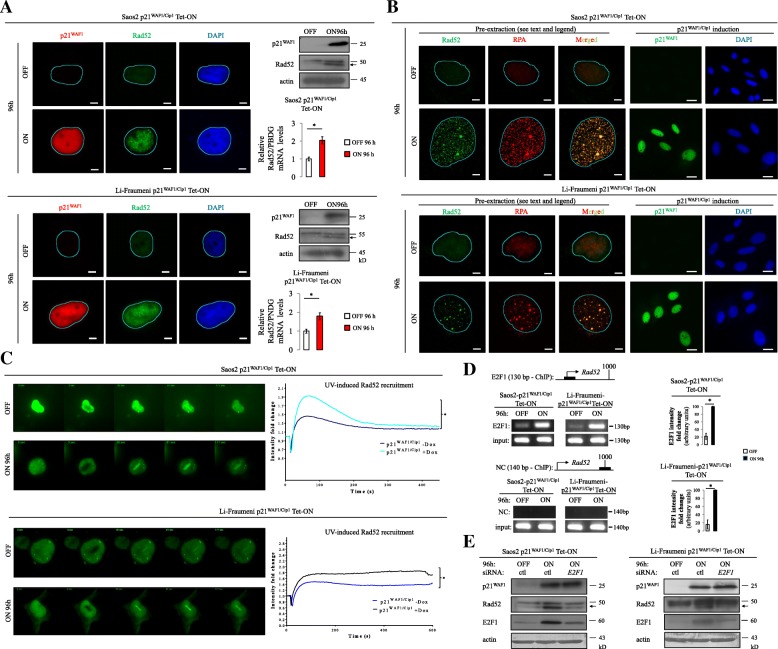
Rad52 increased expression, recruitment, and foci formation at DSBs upon prolonged p21^WAF1/Cip1^ expression. **a** Immunofluorescent (IF) analysis showing increased Rad52 foci formation in 96-h induced Saos2- and Li-Fraumeni-p21^WAF1/Cip1^ Tet-ON cells. Immunoblot (IB) and real-time RT-PCR assessment of Rad 52 expression levels in induced Saos2- and Li-Fraumeni-p21^WAF1/Cip1^ Tet-ON cells at the indicated time point (*p < 0.05 (Saos2), *p = 0.05 (Li-Fraumeni), *t*-test; error bars indicate standard deviation; *n* = 3 experiments). **b** IF analysis showing Rad52 and RPA foci formation and their co-localization in 96-h induced Saos2- and Li-Fraumeni-p21^WAF1/Cip1^ Tet-ON cells. Saos2- and Li-Fraumeni-p21^WAF1/Cip1^ Tet-ON cells were pre-extracted with ice-cold PBS containing 0.2% Triton X-100 for 2 min on ice before fixation as previously described [52]. **c** Timelapse microscopy showing Rad52 loading on regions of damaged chromatin after UV-laser ablation in 96-h induced Saos2- and Li-Fraumeni p21^WAF1/Cip1^ Tet-ON cells transfected with the YFP-Rad52 vector. Plots depict Rad52 recruitment kinetics at sites of DNA damage in the same cells, respectively. The average intensity of fluorescence at the site of damage and the total cell fluorescence in respect to time were quantified and plotted. Five cells in each condition of two independent experiments were processed. **d** *Rad52* promoter is occupied by E2F1 upon p21^WAF1/Cip1^ induction in Saos2- and Li-Fraumeni-p21^WAF1/Cip1^ Tet-ON cells, respectively, as assessed by chromatin immunoprecipitation (ChIP; *p < 0.05, *t*-test; error bars indicate standard deviation; *n* = 3 experiments; see also Additional file 1: Figure S8). **e** Silencing of *E2F1* resulted in decreased Rad52 levels as assessed by immunoblot analysis in induced Saos2- and Li-Fraumeni-p21^WAF1/Cip1^ Tet-ON cells, respectively (*p < 0.01, t-test; error bars indicate standard deviation; *n* = 3 experiments). Actin serves as loading control; (*Ctl*) siRNA; *arrows* indicate Rad52

Next we investigated whether mutational signatures (Fig. 4a; Additional file 1: Figure S1) could guide us to identify potential repair pathway dysfunction. The p21^WAF1/Cip1^ escaped cells demonstrated a similar mutational pattern, in both experimental settings, comprised of SNSs and INDELs, frequently clustered adjacent to breakpoints that exhibited microhomologies (Fig. 4a, b). Interestingly, down-regulation of both *BRCA1* and *BRCA2*, two critical components of homologous recombination (Additional file 1: Figure S6a) with concurrent loss of heterozygosity at the *BRCA2* locus, was also observed (Fig. 4c, d). Comparing the above mutational pattern with the 21 signatures reported by Alexandrov and colleagues [8] we concluded that it is a novel, unique one bearing certain similarities to *signature 3. Signature 3* is characterized by various SNSs along with deletions and insertions of up to 50-bp stretches of DNA with microhomologies at breakpoint junctions and inactivating mutations in *BRCA1* and *BRCA2* [8]*.* The “BRCAness” environment [30], following p21^WAF1/Cip1^ induction, along with the fact that Rad51 recombinase expression was reduced [9], leaves Rad52 recombinase alone to drive Rad51-independent strand-annealing. Consistent with this notion, a strong endogenous Rad52 nuclear immunofluorescence signal was observed in p21^WAF1/Cip1^-induced cells (Additional file 1: Figure S7), reflecting foci formation that co-localized with RPA (Fig. 5a, b), suggesting a shift to Rad52-dependent recombination mechanisms [9]. In concordance with these findings we recorded a fast recruitment and maintenance of Rad52 at sites of DNA damage induced after UV irradiation ablation (Fig. 5c; Additional files 7, 8, 9, and 10).

Intriguingly, the *RAD52* locus was deprived of SNSs and RAD52 protein expression was increased (Fig. 5a). Notably, high protein levels were accompanied by increased transcription of Rad52, which is another interesting observation since most DNA repair factors are upregulated upon DNA damage by post-transcriptional protein modifications that are faster compared to transcriptional control [31]. To obtain a mechanistic insight into Rad52 transcriptional up-regulation, we conducted a bioinformatic analysis of its promoter. We found that the promoter sequence contains binding sites for several transcriptional factors, including that for E2F1 (Additional file 1: Figure S8). Notably, we previously showed that E2F1 is upregulated upon p21^WAF1/Cip1^expression, implying a putative functional link between E2F1 and Rad52 [9]. The functionality of this potential signaling axis was supported by chromatin immunoprecipitation (ChIP) analysis of the *Rad52* promoter showing strong E2F1 binding in p21^WAF1/Cip1^-induced cells (Fig. 5d). Indeed, silencing of *E2F1* led to reduced *Rad52* expression, further supporting the above scenario (Fig. 5e).

In yeast, Rad52 is considered the lynchpin of homologous recombination by facilitating loading of Rad51 on single-stranded DNA (ssDNA) formed through DSB end resection; then Rad51-coated DNA invades the sister chromatid searching for homologous sequences forming D-loop structures (Additional file 1: Figure S6a) [32–34]. However, in mammals, even though Rad52 retains strand-annealing activity [35, 36], Rad51 loading is mediated primarily by BRCA2 [37–39], implying that Rad52 may act as a back-up mechanism. This may explain why organismal development is unaffected in *RAD52*^*−/−*^ mice [40, 41].

**Fig. 6:**
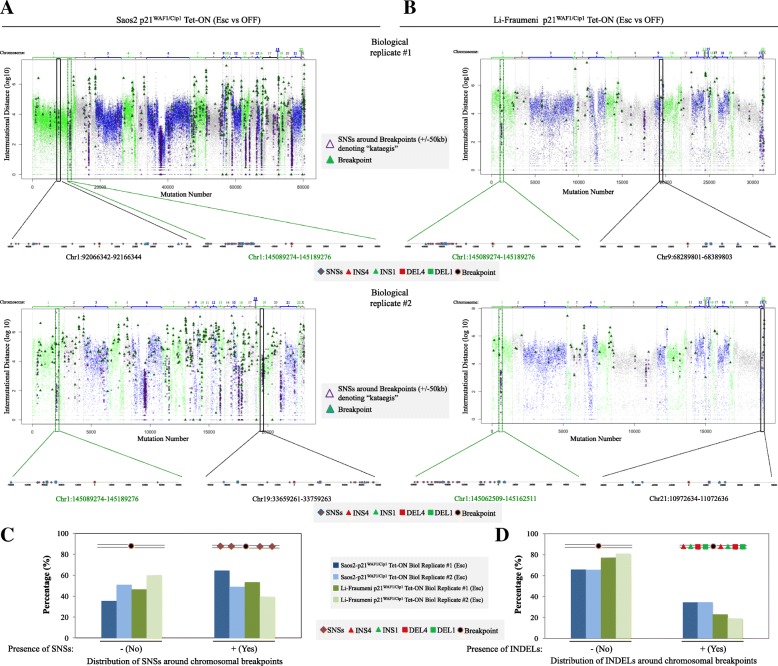
Single nucleotide substitutions (SNSs) cluster around chromosomal breakpoints in cells with continuous p21^WAF1/Cip1^ expression. **a, b** Diagrams depict a dense distribution of SNSs (*purple triangles*) in genome areas surrounding chromosomal breakpoints (*green triangles*), suggestive of the kataegis phenomenon (intense vertically lined piles of SNSs denoted by the *purple triangles*), relative to disparate distribution of SNSs in the remaining genome of escaped (Esc-30 days induced), Saos2- (**a**) and Li-Fraumeni- (**b**) p21^WAF1/Cip1^ Tet-ON cells. Note that the total number of chromosomal breakpoints (*green triangles*) depicted is double as each side of a break corresponds to a different chromosomal arm (Additional file 6: Table S2). The dashed genome areas are depicted as magnifications of representative breakpoints. *Green colored dashed areas* signify representative breakpoints that show a high positional conservation in all experimental (biological) repetitions. Position of breaks and distribution of SNSs, nucleotide insertions (*INS*) and nucleotide deletions (*DEL*) in these examples is depicted in the corresponding subchromosomal magnifications. **c, d** Histograms depict the clustering frequency of SNSs (**c**) as well as INS and DEL (**d**) over all breakpoints in the genome of escaped (Esc-30 days induced) Saos2- and Li-Fraumeni-p21^WAF1/Cip1^ Tet-ON cells (see also Additional file 6: Table S2)

Subsequently, analyzing the distribution of SNSs guided us in understanding which RAD52-dependent repair process took place in the p21^WAF1/Cip1^-induced cells. Particularly, although at the genome-wide level the amount of SNSs was reduced in the escaped cells, the SNSs were interestingly clustered at the flanking regions in a number of novel breakpoint junctions (Fig. 6a–c; Additional file 6: Table S2). This pattern of SNS clustering is termed “kataegis” (Fig. 6a, b) and it requires extensive tracts of ssDNA that act as a substrate for cytidine deamination (Additional file 1: Figure S6a). The later is mediated by the APOBEC family of enzymes leading to C∙G → T∙A transitions and/or C∙G → G∙C transversions [7, 42]. Break-induced replication (BIR), a homologous recombination (HR)-based repair route that allows replication re-start from collapsed replication forks [43], forms such long ssDNA tracts, representing a candidate to repair the p21^WAF1/Cip1^-induced DSBs [9, 44]. During BIR a D-loop is formed followed by a replication fork at the one-ended DNA DSB. The D-loop is not dissolved, but moves together with the fork (migrating bubble) [45, 46], while DNA replication takes place in a conservative manner [47] (Additional file 1: Figure S6a). In yeast, Rad52 seems to play a role in BIR [48], but its role in mammals has just started being elucidated [34, 49, 50]. Nevertheless, not all breakpoints in the escaped cells were flanked by clustered SNSs (Fig. 6c, d; Additional file 6: Table S2), implying that more than one repair pathway is likely involved in processing the p21^WAF1/Cip1^-induced DSBs. Since a high frequency of microhomologies was observed in all novel breakpoints [9], we reasoned that a putative alternative repair route could be single strand annealing (SSA). SSA mediates annealing between two ssDNA ends containing homologous or microhomologous repeats, whereas the 3′ overhanging ends of the processed DSBs are trimmed by XPF-ERCC1 endonuclease [51], depriving the APOBEC editing enzymes from a single strand substrate for cytidine deamination (Additional file 1: Figure S6b). Recently, it was shown that cells deficient in BRCA1 and 53BP1 relied on Rad52-dependent SSA to survive [52].

**Fig. 7:**
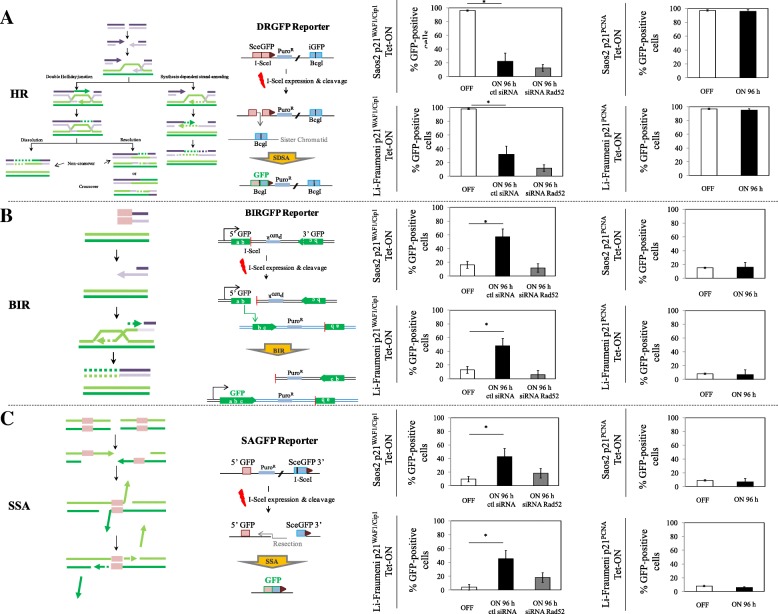
Prolonged p21^WAF1/Cip1^ expression promotes Rad52-dependent break-induced replication (BIR) and single strand annealing (SSA) repair of DNA double strand breaks (DSBs). **a** Reduced synthesis-dependent strand annealing (SDSA) in 96-h induced Saos2- and Li-Fraumeni-p21^WAF1/Cip1^Tet-ON cells. Flow cytometry analysis (FACS) after p21^WAF1/Cip1^ induction in cells stably expressing a DR-GFP report vector and following I-SceI-induced DSBs shows decreased SDSA activity (*p < 0.05, *t*-test; error bars indicate standard deviation; *n* = 5 experiments), regardless of Rad52 silencing. Similar manipulations in Saos2- and Li-Fraumeni-p21^PCNA^ Tet-ON cells showed no differences in SDSA in these cells. **b** Increased BIR activity in 96-h induced Saos2- and Li-Fraumeni-p21^WAF1/Cip1^ Tet-ON cells. FACS after p21^WAF1/Cip1^ induction in cells stably expressing a BIR-GFP report vector and following I-SceI-induced DSBs shows increased BIR activity (*p < 0.05, *t*-test; error bars indicate standard deviation; *n* = 5 experiments) that is suppressed upon Rad52 silencing. Similar experiment in Saos2- and Li-Fraumeni-p21^PCNA^ Tet-ON cells showed no effect on BIR function in these cells. **c** Increased SSA activity in 96-h induced Saos2- and Li-Fraumeni-p21^WAF1/Cip1^ Tet-ON cells. FACS after p21^WAF1/Cip1^ induction in cells stably expressing an SA-GFP report vector and following I-SceI-induced DSBs shows increased SSA activity (*p < 0.05, *t*-test; error bars indicate standard deviation; *n* = 5 experiments) that is dependent on Rad52. A similar experiment in Saos2- and Li-Fraumeni-p21^PCNA^ Tet-ON cells showed no effect on SSA function in these cells

To test the above assumption we monitored DSB repair using GFP reporters, in which DSBs were generated by the nuclease I-SceI. The GFP reporters were stably expressed in the Saos2 and Li-Fraumeni p21^WAF1/Cip1^-inducible systems, and were genetically modified in a way to monitor the main homology-dependent DSB repair modes: gene conversion (GC) and particularly synthesis-dependent strand annealing (SDSA), BIR, and SSA [43, 53] (Fig. 7). DSB formation by I-SceI was followed by protracted p21^WAF1/Cip1^ expression for a period of 4 days. We observed that after day 4 the cells showed stronger fluorescence signals from the GFP reporters monitoring BIR and SSA compared to the control p21^WAF1/Cip1^-OFF cells (Fig. 7b, c), in contrast with the reduced fluorescence read-out from the cells expressing the GFP-SDSA reporter (Fig. 7a). The latter result was also consistent with the decreased expression of key components of the SDSA repair route seen after p21^WAF1/Cip1^ expression (Fig. 4c; Additional file 1: Figure S6a). Notably, exploiting inducible p21^PCNA^ mutant models that cannot interact with PCNA, we noticed that BIR- and SSA-driven repair remained unaffected (Fig. 7b, c), confirming that the altered repair pattern observed in cells with induced wild-type p21^WAF1/Cip1^ was dependent on cellular effects mediated by the interplay of p21^WAF1/Cip1^ with PCNA during DNA replication (Fig. 7a–c). Consistent with such an overall model was also the reduced 53BP1 loading at sites of UV-induced DNA damage (Fig. 1e). 53BP1 fosters homology-directed DNA repair fidelity, but its level in cells is rate limiting, and its exhaustion signifies a shift to the error-prone SSA mechanism [52]. Collectively, these findings support our working hypothesis, and given the low levels of Rad51 and BRCA2, the data furthermore demonstrate the inability of SDSA to deal with p21^WAF1/Cip1^-triggered DSBs. To test the cause–effect relationship, we silenced *Rad52* to examine the dependency of BIR and SSA on Rad52, predicted by our model. Indeed, depletion of Rad52 led to a significant suppression of GFP fluorescence readouts from p21^WAF1/Cip1^-induced cells harboring both the BIR- and SSA-GFP repair reporters (Fig. 7b, c), thereby further supporting the requirement for Rad52 in these repair pathways in our experimental settings [49, 52].

## Discussion

Genome maintenance is a fundamental prerequisite for preserving cellular life, under both physiological and pathological conditions. Even the highly unstable aberrant cancer genomes must be maintained within certain limits of genomic integrity, beyond which cells would die. Better understanding of the molecular mechanisms that allow genome destabilization that fuels cancer development and progression yet protect the cancer genome from too severe, fatal instability is vital for the development of new therapeutic strategies [6]. Replication stress (RS) driven-genomic instability emerges as a major force promoting cancer evolution [4, 54, 55]. To identify the DNA repair pathways that help cancer cells adapt and survive under such chronic stress is key to both understanding tumorigenesis and finding clinically exploitable vulnerabilities of tumor cells.

Based on the results obtained in this study, we propose a concept whereby chronic expression of p21^WAF1/Cip1^induces a dramatic rewiring of the cellular DNA repair pathway choices, providing further means in conjunction with deregulated replication to fuel genomic instability. Using human cellular models with inducible expression of p21^WAF1/Cip1^ that evokes replication stress [9], we now show that such a scenario leads to suppression of the TLS-mediated repair, with ensuing defective processing of single nucleotide lesions, eventually resulting in replication fork stalling and collapse, generating DNA DSBs. Having in mind that most human cancers harbor p53 and p16^INK4^/pRb alterations [56], p53-independent expression of p21^WAF1/Cip1^ is one of the few remaining cellular guardians against the accumulating (pro)tumorigenic insults. Under such circumstances, p21^WAF1/Cip1^-induced cellular senescence is often reversible and during this temporary anti-tumor response dramatic chromosomal remodeling takes place that favors over time the birth of aggressive offspring [9]. Here we provide mechanistic insights into the error-prone repair process that occurs during this genome-destabilizing evolutionary trajectory, by demonstrating that p21^WAF1/Cip1^ induced DSBs are commonly repaired by Rad52-dependent break-induced replication (BIR) and single strand annealing (SSA), as the synthesis-dependent strand annealing (SDSA) repair route was defected (Figs. 5 and 7; Additional file 1: Figure S6). BIR is a highly error-prone homologous recombination (HR)-response that has been implicated in formation of high-frequency tandem segmental duplications found in cancer [7, 43]. Moreover, BIR introduces mutations in the newly synthesized DNA strand at a much higher rate than under conditions of conventional replication [44, 57]. Likewise, SSA is associated with extensive DNA resection contributing to genome rearrangements and oncogenic transformation [58].

Furthermore, we show that Rad52 was upregulated transcriptionally in a manner dependent on E2F1, a transcription factor that also drives G1/S transition and which is frequently overexpressed in cancer [59]. As the RB pathway is almost universally deregulated in tumors, E2F1 is often free from the restraining binding to pRB and hence capable of inducing Rad52. This, in turn, compensates for the reduced levels of Rad51 which is in short supply under stressful conditions [60]. Transcriptional regulation, under genotoxic stress, as seen here for Rad52, was unexpected since most DNA damage response and repair proteins become rapidly upregulated via post-translational modifications that slow down the protein turnover [31]. Given the low levels of pivotal components (Rad51, BRCA1, and BRCA2) of the SDSA repair machinery (Fig. 4c, d; Additional file 1: Figure S6a), the p21^WAF1/Cip1^ expressing cells resort to transcriptional upregulation of Rad52, a fact that reflects the increased repair needs required to cope with the p21^WAF1/Cip1^-driven replication stress [61]*.* On the other hand, post-translational modifications of the chromatin bound fraction of Rad52 cannot be excluded, as recently reported [49], and could further contribute to increased abundance of Rad52.

In contrast to yeast, where Rad52 represents a major factor in the first line of genome maintenance [33], in higher eukaryotes and mammals Rad52 seems to serve as a reserve player that can substitute for other repair options when those are compromised. Overall, this concept highlights Rad52 as a potential therapeutic target in tumors with inactive BRCA2 and helps explain why *Rad52* gene amplifications are selected for in human cancers [62–64]. Thus, targeting Rad52 could turn out to be a new way to therapeutically exploit vulnerabilities that occur selectively in cancer cells. Consistent with this idea, depletion of *Rad52* confers synthetic lethality in *BRCA2* deficient cells [65, 66].

## Conclusions

**Fig. 8:**
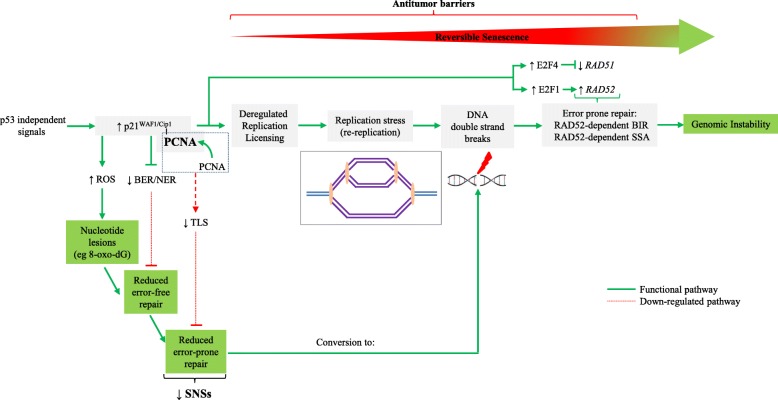
Proposed model depicting how p53-independent p21^WAF1/Cip1^ expression fuels Rad52-dependent error-prone double strand break repair promoting genomic instability. Sustained p53-independent p21^WAF1/Cip1^ induction leads to increased levels of nucleotide lesions mediated by elevated reactive oxygen species (ROS). Given the negative impact exerted by p21^WAF1/Cip1^ on the error free nucleotide repair mechanisms (BER and NER), a significant proportion of such base lesions escape unrepaired. This creates an additional repair “load” to the error prone repair mechanism of TLS, which is further compromised by p21^WAF1/Cip1^ overexpression, leading to a decreased SNS load and in favor of DSBs. In turn, this further increases the DSB burden generated also through re-replication [9]. As components of SDSA are down-regulated, a shift to Rad52-mediated error prone DNA repair takes place by invoking the BIR and SSA repair routes, fueling genomic instability. This repair switch is mediated by a shift in the balance between Rad51 and Rad52 levels as the former is suppressed by E2F4 [9] and the latter is induced by E2F1 (present study). *DSB* DNA double strand break, *BER* base excision repair, *NER* nucleotide excision repair, *TLS* translesion DNA synthesis and repair, *SDSA* synthesis-dependent strand annealing, *BIR* break-induced repair, *SSA* single strand annealing

On the whole, the current study broadens our understanding of how chronic p53-independent p21^WAF1/Cip1^ expression, seen in a sizeable fraction of advanced human tumors [9], impacts the global DNA repair landscape and undermines genomic stability. The salient features of our model are the following: i) saturation of the CRL4^CDT2^ligase complex by p21^WAF1/Cip1^ impairs the turn-over of the replication licensing factors leading to their unscheduled accumulation, causing ii) genome re-replication and replication stress, as recently described [9], while iii) concurrent suppression of the DNA damage tolerance (TLS) pathway reduces the repair rate of the nucleotide lesions in an environment with dysfunctional error-free excision repair mechanisms of BER, NER, and MMR. As a consequence of such grossly rewired DNA repair pathway choice, the rate of unrepaired nucleotide lesions increases, further raising the burden on the already limited TLS. Both features of DNA re-replication-induced replication stress and deficient TLS lead to enhanced formation of the highly deleterious DSBs that are repaired in an error-prone manner, by Rad52-dependent BIR and SSA, thereby fueling genomic instability and promoting cancer development (Fig. 8). We hope the concept proposed here may not only inspire further mechanistic studies, but also attempts to target Rad52 in cancer, as a way to selectively induce lethal chromosomal instability in Rad52-dependent cancers, while sparing normal tissues whose genome maintenance does not depend on Rad52. Lastly, the present study underscores the significance of identifying mutational signatures as they can unveil the repair procedure(s) that fuel genomic instability and thus highlight potential therapeutic targets for cancer treatment.

## Methods

### Cell lines and culture conditions

Inducible p21^WAF1/Cip1^ Tet-ON cell lines—Saos2- p21^WAF1/Cip1^ Tet-ON and Li Fraumeni- p21^WAF1/Cip1^ Tet-ON—were maintained in High Glucose DMEM (Biosera) supplemented with 10 % Tet System Approved FBS (Clontech) and 100 μg/ml penicillin and streptomycin (Invitrogen) and incubated at 37 °C and 5 % CO_2_. Induction of p21^WAF1/Cip1^ was conducted by treatment of the cell culture with 1 μg/ml doxocycline (Applichem) [9].

### siRNA and vector transfections

Rad52 (Thermo Scientific) siRNA gene silencing was performed as previously described, following the manufacturer’s instructions [9]. YFP-RAD52 [52] and Polκ vectors were transfected as previously described [67].

### Protein extraction, cell fractionation, and immunoblotting

Protein extraction and cell fractionation were performed as described before [9]. Thirty micrograms of protein from total extracts per sample were adjusted with Laemmli buffer (Sigma) and loaded on acrylamide/bis-acrylamide gels. Gel electrophoresis, transfer to PVDF membrane (Millipore), and signal development with nitro blue tetrazolium/5-bromo-4-chloro-3-indolylphosphate (NBT/BCIP) solution (Molecular Probes) or chemiluminescence were performed as previously described [9]. Alkaline phosphatase-conjugated anti-mouse or anti-rabbit as well as horse radish peroxidase conjugated anti-mouse, anti-rabbit, and anti-sheep secondary antibodies (1:1000 dilution; Cell Signaling) were used.

Primary antibodies utilized were anti-p21^WAF1/Cip1^ (mouse, Santa Cruz, sc-6246, 1:400 for IB), anti-RAD52 (mouse, Santa Cruz, sc-365,341, 1:100 for IF), anti-β-actin (rabbit, Cell Signaling Tech, 4967 s, 1:1000 for IB), anti-PCNA (mouse, Cell Signaling Tech, 2586 s, 1:1000 for IB), anti-Ubiquityl-PCNA (rabbit, Cell Signaling Tech, 13,439 s, 1:1000 for IB), anti-polη (rabbit, Santa Cruz, sc5592, 1:200 for IB), anti-BRCA1 (mouse, SantaCruz, sc6954, 1:200 for IB), anti-BRCA2 (mouse, Merck Millipore, OP95, 1:200 for IB), anti-DDB1 (mouse, Abcam, ab13562, 1:1000 for IB), anti-LIG3 (rabbit, Abcam, ab185815, 1:1000 for IB), anti-APEX1 (mouse, Abcam, ab194, 1:2000 for IB), anti-TDG (mouse, Novus Biologicals, NBP2–43717, 1:5000 for IB), anti-MUTY (rabbit, Thermo Fisher Scientific, PA5–26167, 1:1000 for IB), anti-ERCC1 (mouse, SantaCruz, sc17809, 1:1000 for IB), anti-ERCC4-XPF (mouse, Thermo Fisher Scientific, MA5-12060, 1:500 for IB), anti-ERCC5-XPG (rabbit, Abcam, ab189317, 1:500 for IB), anti-XPC (mouse, Santa Cruz, sc-74,410, 1:500 for IB), anti-XRCC1 (mouse, Abcam, ab1838, 1:200 for IB), anti-NEIL2 (rabbit, Abcam, ab124106, 1:500 for IB), anti-α-tubulin (mouse, GeneTex, GTX628802, 1:5000 for IB), anti-β-tubulin (rabbit, Abcam, ab6046, 1:1000 for IB), anti-lamin B1 (rabbit, Abcam, ab16048, 1:1000 for IB). All analyses were performed in triplicate.

### Indirect immunofluorescence

Indirect immunofluorescence analysis was performed as previously published [9]. Regarding identification of RAD52 foci, parameters of the aforementioned process have been set as indicated by Ochs and collaborators [52] and by Sotiriou and collaborators [49]. For all RPA and Rad52 IF co-localization experiments (Fig. 5b) Saos2- and Li-Fraumeni-p21^WAF1/Cip1^ Tet-ON cells were pre-extracted with ice-cold PBS containing 0.2% Triton X-100 for 2 min on ice before fixation as previously described [52]. Secondary antibodies were Alexa Fluor 488 donkey anti-sheep (Abcam, ab150177, 1:500) and Alexa Fluor 568 goat anti-mouse (Invitrogen, no. A11031, 1:500). Image acquisition of multiple random fields was automated on a DM 6000 CFS Upright Microscope (Confocal Leica TCS SP5 II) or a ScanR screening station (Olympus) and analyzed with ScanR (Olympus) software, or a Zeiss Axiolab fluorescence microscope equipped with a Zeiss Axiocam MRm camera and Achroplan objectives, while image acquisition was performed with AxioVision software 4.7.1. Primary antibodies utilized were anti-p21^WAF1/Cip1^ (mouse, Santa Cruz, sc-6246, 1:200 for IF), anti-RAD52 (sheep [52], 1:100 for IF and mouse, Santa Cruz, sc-365,341, 1:100 for IF). All analyses were performed in triplicate.

### Image acquisition

Live DNA damage protein recruitment kinetics were observed using an Olympus IX83 inverted microscope system. DNA irradiation damage was induced on the same system using a coupled UVA (355 nm) pulsed laser (teemphotonics PNV-M02510) and a theoretical pulse duration of less than 350 psec. Subnuclear irradiations were performed on an 8-μm linear ROI with the use of a total of 60–90 pulses subdivided into three repeats, with 2.3 % of the total laser power. Pulse irradiation calibration was titrated through γH2Ax post damage spatial organization on MCF7 cancer cells as previously described [68]. For time-lapse acquisition an Olympus Apochromat 63×/ 1.2NA water immersion lens and a Hamamatsu ORCA Flash 4.0. sCMOS camera system were used. The microscope was equipped with a temperature/humidity and CO_2_ incubation system (CellVivo) and with a 6-LED system (Lumencor) as light source.

Brief powerful laser ablation inscribed cell location within the glass volume of the coverslip below the cells of interest. This technique enables easy location of the marked fields of view on any microscope under transmission contrast.

Z-stack imaging was conducted on a Leica SP5 TCS equipped with a hybrid detector and a 60×/1.4NA oil immersion lens. A z-step of 0.72 μm was used and a total of 18 stacks were obtained per nucleus.

#### Cell culture

For live-cell experiments, cancer cells were plated on Ibidi glass bottom dishes (idibi μ-dish 35 mm 81,156) in phenol red-free, Minimum Essential Medium Eagle (MEM). L-glutamine 2 mM, hepes 25 mM final concentration, and 10 % fetal bovine serum (FBS) were added.

#### Image analysis

Kinetics analyses were quantified using Fiji distribution of ImageJ (v 2.0.0-rc-30/1.49 s). Images were background corrected by subtracting the mean intensity value of an area outside the cell (ROI3). Values of corrected mean intensity of the site of damage (RO1’) and the corrected total fluorescent-area mean intensity were obtained at each time point (ROI2’). Cells were normalized for the different protein expression levels and acquisition photobleaching with the use of this formula:

$$ \boldsymbol{I}{\left(\boldsymbol{t}\right)}_{norm}^{double}=\left(\frac{\frac{1}{{\boldsymbol{n}}_{pre}}\cdot \sum \limits_{i=1}^{n_{pre}}\boldsymbol{I}{\left(\boldsymbol{t}\right)}_{ROI{2}^{\prime }}}{\boldsymbol{I}{\left(\boldsymbol{t}\right)}_{ROI{2}^{\prime }}}\right)\cdot \left(\frac{\boldsymbol{I}{\left(\boldsymbol{t}\right)}_{ROI{1}^{\prime }}}{\frac{1}{{\boldsymbol{n}}_{pre}}\cdot \sum \limits_{t=1}^{n_{pre}}\boldsymbol{I}{\left(\boldsymbol{t}\right)}_{ROI{1}^{\prime }}}\right) $$

### ChIP assay

ChIP assay was performed as previously described [67]. A 130-bp fragment in the *Rad52* promoter and a 140-bp amplicon, located approximately 1000 bp from the transcription start site (Fig. 5d), were amplified. Primers and annealing temperatures are provided in Additional file 6: Table S3. PCR reactions containing 1 % of the total chromatin extract used in the immunoprecipitation reactions were used as inputs. Three independent assays were performed.

### TLS assay

Cells were co-transfected with a plasmid mixture containing the gap-lesion plasmid (kan^R^), a control gapped plasmid without a lesion (cm^R^), and the carrier plasmid pUC18 (amp^R^). After allowing time for gap filling and lesion bypass, plasmids were extracted using alkali, such that only filled-in plasmids remained intact. To assay the fraction of filled-in plasmids, the plasmid mixture was transformed into an indicator *Escherichia coli recA* strain and plated in parallel, on LB-kan plates (to select for plasmids that underwent TLS) and LB-cm plates (to select for the control filled-in plasmid GP20-*cm*). TLS in this case was calculated by the ratio of kan^R^/cm^R^*E. coli* transformants. Specifically, the cells were co-transfected with a DNA mixture containing 100 ng of a gap-lesion plasmid (GP BP-G; kan^R^), 100 ng of a gapped plasmid without lesion (GP20-*cm*, cm^R^), and 2300 ng of the carrier plasmid pUC18, using Lipofectamine®2000/DNA complexes. The percentage of lesion bypass gap filling was calculated by dividing the number of GP BP-G transformants (number of colonies on LB-kan plates) by the number of corresponding GP20-cm transformants (number of colonies on LB-cm plates). When desired, plasmids were extracted from kan^R^ colonies, and the sequence opposite the lesion was determined by DNA sequence analysis [18].

### Comet assay

In order to determine the endogenous (background) levels of oxidatively induced DNA damage we performed the sensitive technique of single cell gel electrophoresis (SCGE; Comet assay) under alkaline (denaturing) conditions as previously described [67]. We also used as a DNA damage probe the human repair enzyme OGG1 (New England Biolabs) to detect the specific presence of 8-oxo-dGuanine (8-oxo-dG) [24]. An increase in the tail moment (TM) suggests higher levels of oxidative DNA damage (primarily oxidized purines). Cells were observed under a Zeiss Axiolab fluorescence microscope equipped with a monochrome CCD camera. Analysis was conducted with Cometscore software (Tritek). All experiments were performed five times.

### Melphalan assay

Melphalan [(4-(bis{2-chloroethyl} amino)-l-phenylalanine] belongs to the nitrogen mustard class of chemotherapeutic agents, used in the treatment of certain hematological maligancies. The monofunctional derivative of melphalan (monohydroxymelphalan) induces only monoadducts, which are almost exclusively repaired by nucleotide excision repair [69–71]. Its mode of action is by alkylating the DNA, generating predominantly N-alkylpurine monoadducts and to a minor extent interstrand cross-links (ICLs), the formation of which are dependent on these monoadducts. These lesions primarily affect the N-7 position of guanines and to a lesser degree the N-3 position of adenines.

Preparation of the monofunctional derivative of melphalan was performed as described previously [69, 70]. Cell lines were treated with monohydroxymelphalan (100 μg/ml, 5 min, 37 °C) in culture medium. Then, cells were incubated in drug-free medium for various times (up to 48 h), harvested, and stored at −70 °C.

To measure the induction and repair of melphalan-derived DNA adducts, a specific assay is applied [26]. In principle, the N-ras-specific nucleotide excision repair was evaluated by the monofunctional binding of monohydroxymelphalan to a single site in the DNA molecule (monoadducts) at various time points as described previously [71]. Briefly, genomic DNA was digested to completion with the restriction enzyme EcoRI and DNA samples dissolved in sterile deionized H_2_O were heated at 70 °C for 30 min to depurinate N-alkylated bases. Apurinic sites were converted to single strand breaks by the addition of NaOH for 30 min at 37 °C, size fractionated using agarose gel electrophoresis, and Southern blotted. Hybridizations were performed as described previously. The average frequency of N-ras-specific monoadducts in the restriction fragment of interest was calculated from the fraction of DNA in the band from the treated sample compared to that from the non-treated sample. To minimize the inaccuracy in the measurement of DNA damage arising from errors in DNA quantification or gel loading, in all experiments an internal standard (part of the N-ras gene) was included. Data were obtained from five independent experiments with two repetitions for each time point in each experimental set.

### 8-Oxo-dG assay

A previously described assay based on the property of avidin to bind with high specificity to 8-oxo-dG was used for the 8-oxoG measurements [24]. Briefly, cells were fixed in methanol at −20 °C for 20 min and incubated for 15 min in TBS, 0.1 % Triton X-100. Blocking was performed in 15 % FBS, 0.1 % Triton X-100 in TBS for 2 h at room temperature (RT). Cells were then incubated with 10 μg/ml Alexa488-conjugated avidin (Invitrogen) in blocking solution for 1 h at 37 °C. Next they were rinsed twice in TBS, 0.1 % Triton X-100 for 5 min each round at room temperature. After a quick rinse in distilled water, DNA was counterstained with ToPro3-Iodide (LifeTechnologies) for 15 min at room temperature, followed by a final rinse in TBS.

Coverslips were mounted with ProLongGold (Invitrogen) and cells were observed under a Zeiss Axiolab fluorescence microscope equipped with a monochrome CCD camera. Analysis was conducted with NIH-imageJ, with respect to mean intensity in the nucleus (To-Pro3 served as a DNA reference). All experiments were repeated five times.

### Measurement of intracellular levels of reactive oxygen species

Intracellular levels of reactive oxygen species (ROS) were calculated using the DCFH-DA assay. In details, the cells were plated in a 96-well plate at a density of 10,000 cells/well and when ~ 80 % confluent they were treated with doxocycline (1 μg/ml) in DMEM supplemented with 10 % (*v*/v) FBS until confluence. At the indicated time points DCFH-DA (10 μM) was added and after a further incubation of 1 h measurements (excitation wavelength 480 nm, emission wavelength 530 nm) were taken in a FLUOStar OPTIMA microplate reader (BMG Labtech GmbH, Ortenberg, Germany) using the MARS Data Analysis Software. After this measurement, the number of cells was estimated and ROS levels were expressed as fluorescence units per cell number [72]. Average data from five independent experiments were obtained.

### cDNA preparation and real-time quantitative PCR with reverse transcription

cDNA generation and real-time quantitative PCR with reverse transcription analysis were performed as described before [9]. The reaction was performed in a StepOne Real time machine (Life Technologies) using Universal MasterMix II without UNG containing SYBR (Life Technologies) and 200 nM primers. Signal analysis was carried out using the StepOne v2.3 software. Primers and annealing temperatures are provided in Additional file 6: Table S3. All analyses were performed in triplicate.

### DR-GFP, SA-GFP, and BIR-GFP reporter assays

Saos2-p21^WAF1/Cip1^ Tet-ON cells harboring the GFP based reporter constructs for synthesis-dependent strand annealing (DR-GFP), single strand annealing (SA-GFP), and break-induced replication (BIR-GFP) were generated by transfection with these DSB repair reporters (Additional file 6: Table S4), followed by selection of stably transfected clones [43, 53]. To monitor the repair of an I-SceI-generated DSB, cells were transiently transfected with 1 μg of the I-SceI expression vector HA-ISceID44A (Addgene #59424) with effectene transfection reagent (Qiagen). DSB repair efficiency, upon induction of p21^WAF1/Cip1^, was determined by quantifying GFP-positive cells via flow cytometry FACS Calibur (Becton Dickinson) 48 h after transfection. Data were obtained from five independent experiments.

### High-throughput whole-genome analyses

#### Whole-genome sequencing analysis

Whole-genome sequencing (WGS) library preparation, alignment, and breakpoint identification were performed as described before [9]. Samtools mpileup and vcftools [73] were used for identification and filtering of the SNSs and INDELs. SNSs and INDELs that were unique in the escaped cells were normalized based on the depth of the sequencing for each experiment. In more detail we generated .bam files with the use of bowtie2 (“--very-sensitive”) and samtools (“view” command). We used the .bam files to extract the SNSs and INDELs with the use of samtools (“mpileup” command) and bcf/vcf tools to convert generated .bcf files to .vcf and separate the SNSs from the INDELs. VCF files containing SNSs and INDELs were filtered based on their mapping quality (MQ ≥ 30) while “genotypes” (GT) (0/1 = 1 allele and 1/1 = both alleles) corresponding to allele frequency greater than 0.5 (AF ≥ 0.5) were kept for downstream analysis. The algorithms for measuring the SNS load and identifying the mutational signatures are schematically presented in Additional file 1: Figure S1 and described as follows.

#### SNS load

The SNS load was calculated after filtering for mapping quality (MQ ≥ 30), allele frequency (AF ≥ 0.5), and sequencing depth (DP) of the total number of SNSs identified with the use of Samtools and VCF tools for each of the eight WGS experiments (*n* = 8). For read depth normalization in the WGS experiment we calculated the coverage of the aligned reads in the effective human genome and selected those SNSs and INDELs with read-depth equal to the average normalized coverage for each experiment.

#### Mutational signature

Mutational signature was performed with the use of the SNSs that were filtered for mapping quality in both escaped (Esc)/non-induced (OFF) cells. After intersecting the SNSs in the escaped cells with the SNSs in the non-induced (OFF) cells applying the bedtool algorithm (“intersectBed”) [74] we used the newly identified SNSs (Esc-specific as shown in Additional file 1: Figure S1), which we filtered for allele frequency and read depth. The newly identified SNSs were employed for generating the mutation signature of the escaped cells. By applying intersection between the two cell populations and then filtering based on read depth and allele frequency we removed any bias due to low coverage in OFF cells and focused on SNSs that appeared only in the escaped cells.

#### RNA-seq analysis

RNA was collected from non-induced and 96-h induced (4 day) Saos2 p21 Tet-ON cells, as well as from non-induced and 96-h induced (4 day) Li-Fraumeni p21 Tet-ON cells (three biological replicates for each condition). RNA-seq library preparation and analysis of 75-bp paired-end reads procedure was performed in the Greek Genome Center (GGC) of Biomedical Research Foundation of Academy of Athens (BRFAA). TopHat2 (2.0.9) [75] was used for data alignment with the use of “- - sensitive” option to the hg19 genome version, while HT-seq count algorithm [76] was used for assigning aligned reads to the human transcriptome. Identification of the differentially expressed genes was performed with R/Bioconductor and DESeq [77] algorithm and genes with absolute fold change ≥ 1.5 and *p* value ≤ 0.05 were considered as differentially expressed between induced and non-induced cells.

## Additional files

Additional file 1: **Figures S1.** to S8 [79–81]. (DOCX 1894 kb)

Additional file 6: **Tables S1.** to S4. (DOCX 71 kb)

## Abbreviations

8-oxo-dG: 8-Oxo-dGuanine
AP sites: Apurinic/apyrimidinic sites
BER: Base excision repair
BIR: Break-induced replication
BPDE: Benzo[*a*]pyrene diol epoxide
DDT: DNA damage tolerance
DSB: Double strand break
GC: Gene conversion
HR: Homologous recombination
MMR: Mismatch repair
NER: Nucleotide excision repair
OGG1: 8-Oxoguanine glycosylase
PRR: Post-replication repair
ROS: Reactive oxygen species
RS: Replication stress
SDSA: Synthesis-dependent strand annealing
SNS: Single nucleotide substitution
SSA: Single strand annealing
TLS: Translesion DNA synthesis
