# Author Correction: Mutational signatures reveal the role of RAD52 in p53-independent p21-driven genomic instability

**DOI:** 10.1186/s13059-022-02678-y

**Published:** 2022-04-28

**Authors:** Panagiotis Galanos, George Pappas, Alexander Polyzos, Athanassios Kotsinas, Ioanna Svolaki, Nickolaos N. Giakoumakis, Christina Glytsou, Ioannis S. Pateras, Umakanta Swain, Vassilis L. Souliotis, Alexandros G. Georgakilas, Nicholas Geacintov, Luca Scorrano, Claudia Lukas, Jiri Lukas, Zvi Livneh, Zoi Lygerou, Dipanjan Chowdhury, Claus Storgaard Sørensen, Jiri Bartek, Vassilis G. Gorgoulis

**Affiliations:** 1 Molecular Carcinogenesis Group, Department of Histology and Embryology, School of Medicine, National Kapodistrian University of Athens, 75 Mikras Asias Str, GR-11527 Athens, Greece; 2 Danish Cancer Society Research Centre, Strandboulevarden 49, DK-2100 Copenhagen, Denmark; 3 Biomedical Research Foundation of the Academy of Athens, 4 Soranou Ephessiou Str, GR-11527 Athens, Greece; 4 Laboratory of Biology, School of Medicine, University of Patras, 26505 Patras, Rio Greece; 5 Department of Biology, University of Padova, 35121 Padova, Italy; 6 Department of Biomolecular Sciences, Weizmann Institute of Science, 76100 Rehovot, Israel; 7 Institute of Biology, Medicinal Chemistry and Biotechnology, National Hellenic Research Foundation, 48 Vassileos Constantinou Ave, GR-11635 Athens, Greece; 8 Physics Department, School of Applied Mathematical and Physical Sciences, National Technical University of Athens (NTUA), 15780 Zografou, Athens Greece; 9 Department of Chemistry, New York University, New York, 10012 USA; 10 Novo Nordisk Foundation Center for Protein Research, Faculty of Health and Medical Sciences, University of Copenhagen, Copenhagen, Denmark; 11 Department of Radiation Oncology, Dana-Farber Cancer Institute, 450 Brookline Ave, Boston, MA 02215 USA; 12 Harvard Medical School, 25 Shattuck St, Boston, MA 02115 USA; 13 Biotech Research and Innovation Centre (BRIC), University of Copenhagen, Ole Maaloes Vej 5, DK-2200 Copenhagen, Denmark; 14 Science for Life Laboratory, Division of Genome Biology, Department of Medical Biochemistry and Biophysics, Karolinska Institute, SE-171 77 Stockholm, Sweden; 15 Faculty of Biology, Medicine and Health, University of Manchester, Manchester Academic Health Science Centre, Wilmslow Road, Manchester, M20 4QL UK

**Author Correction: Genome Biol 19, 37 (2018)**

**https://doi.org/10.1186/s13059-018-1401-9**

Following publication of the original article [1], the authors identified an error in additional file 1. Two minor typing errors were discovered in legends S2 and S6. The updated additional file 1 is given in this correction article.

## Supplementary Information

**Additional file 1.** Figures S1. to S8 [79–81].
