# Supplementary material for: Mutational signatures reveal the role of RAD52 in p53-independent p21-driven genomic instability
Source: Genome Biol. 2018 Mar 16;19:37. doi: 10.1186/s13059-018-1401-9 (PMC5857109; doi:10.1186/s13059-018-1401-9)
Supplement: Supplementary file 9 — Figures S1. to S8 [79–81]. (DOCX 1894 kb) [file 13059_2018_1401_MOESM1_ESM.docx]

**ADDITIONAL FILE 1: Figures S1 to S8**

**Mutational signatures reveal the role of RAD52 in p53-independent p21 driven genomic instability**

Panagiotis Galanos^1,2*^, George Pappas^1,2*^, Alexander Polyzos^3^, Athanassios Kotsinas^1^, Ioanna Svolaki^1^, Nickolaos N Giakoumakis^4^, Christina Glytsou^5^, Ioannis S Pateras^1^, Umakanta Swain^6^, Vassilis L Souliotis^7^, Alexandros G Georgakilas^8^, Nicholas Geacintov^9^, Luca Scorrano^5^, Claudia Lukas^10^, Jiri Lukas^10^, Zvi Livneh^6^, Zoi Lygerou^4^, Dipanjan Chowdhury^11,12^, Claus Storgaard Sørensen^13^, Jiri Bartek^2,14**^, Vassilis G. Gorgoulis^1,3,15**^

* Authors equally contributed

** To whom correspondence should be addressed:

**Vassilis G. Gorgoulis,** E-mail: vgorg@med.uoa.gr; or: vgorgoulis@gmail.com;

Tel.: 0030-2107462352 **and** **Jiri Bartek**, E-mail: [jb@cancer.dk](mailto:jb@cancer.dk); Tel.: +45 – 35257357

**This file includes:**

Figures S1 to S8

**Figure S1. SNS load and mutational signature estimation algorithms in non-induced and “escaped” Saos2- and Li-Fraumeni-p21^WAF1/Cip1^ Tet-ON cells.** Four independent experiments, twice for each cellular system Saos2- and Li-Fraumeni-p21^WAF1/Cip1^ Tet-ON cells, respectively, were deployed at different chronological time points (top graph). The OFF cells were cultured once the subpopulation of “escaped” cells emerged (left graphs). In this manner, parallel growth of the non-induced (OFF) cells during the senescent phase that could lead to further accumulation of SNSs was avoided. Different aliquots of same clones were used in each experiment for the same cellular system. Methodology employed to estimate SNS load and mutational signature in non-induced (OFF) and “escaped” cells (see details in Materials-Methods section).

**Figure S2. Nucleotide lesions repair hypothesis under protracted p21^WAF1/Cip1^ expression.** **(a)** *Efficient high fidelity (error-free) repair.* Under normal “ideal” conditions the high fidelity BER and NER repair mechanisms [**22, 25**] deal with the nucleotide lesions. **(b)** *Deficient high fidelity (error-free) repair leading to low fidelity TLS repair.* Increased accumulation of nucleotide lesions overwhelming the high fidelity repair mechanisms capacity and/or defects in these error-free repairing mechanisms lead to TLS engagement for nucleotide lesion repair [**11**]. This should result in increased single nucleotide substitution (SNSs) levels. **(c)** *Deficient high fidelity (error-free) and low fidelity TLS repair.* Increased accumulation of nucleotide lesions overwhelming the high fidelity repair mechanisms capacity and/or defects in these error-free repairing mechanisms combined with TLS deficiency result in unrepaired nucleotide lesions. Instead of being corrected in an error-prone manner (reduced SNS accumulation) they cause replication fork stalling, collapse, eventually leading to DNA double strand breaks (DSBs), mutagenic DSBs repair and gross chromosomal rearrangemenrs (GCRs).

**Figure S3.** **Reduced expression of components of the Base Excision Repair (BER), Nucleotide Excision Repair (NER) and Mismatch Repair (MMR) pathways in cells with sustained p21^WAF1/Cip1^ expression.** (**a, c**) Expression status of components of the BER, NER and MMR pathways as assessed by real-time RT-PCR in 96h induced Saos2- and Li-Fraumeni-p21^WAF1/Cip1^ Tet-ON cells, validating the high-throughput expression results (see also **Figs. 2, 3**) (*p* < 0.01, t-test, error bars indicate SDs, n=3 experiments). Note that for OGG1 expression status, data from previous microarray RNA analysis in Saos2-p21^PCNA^ Tet-ON cells was also available [**9**]. (**b, d**) RNAseq analysis of essential factors of the MMR pathway in 96h induced Saos2- and Li-Fraumeni-p21^WAF1/Cip1^ Tet-ON cells. The lower right panels depict the components and steps implicated during MMR. The MMR pathway is responsible for recognition and repair of mismatched bases generated due to misincorporation during DNA replication. In eukaryotes the mechanism of MMR involves the initial recognition of the mismatched base by MutSα/β, followed by incision from the endonuclease activity of MutLα of the 3’- or 5’- side of the mismatched base on the discontinuous strand. Subsequently, EXO I exonuclease excises the resulting DNA segment, in cooperation with the single-stranded DNA-binding protein RPA. The resected DNA strand is resynthesized by DNA polymerase δ and DNA Ligase. [MSH2/3/6: MutS homolog 2/3/6; MLH1/3: MutL homolog 1/3; PMS1/2: PMS1/2 homolog 1, mismatch repair system component; EXO1: Exonuclease 1]

**Figure S4. Differentially expressed components of the Base Excision Repair (BER), and Nucleotide Excision Repair (NER) pathways in cells with protracted p21^WAF1/Cip1^ expression.** (**a**) Differentially expressed genes in BER at mRNA (i) and protein level (ii) in the Saos2- and Li-Fraumeni-p21^WAF1/Cip1^ Tet-ON cells. Note one exception, namely XRCC1 that showed similar protein levels in the two cellular systems, despite the differential mRNA levels. Immunoblots were perfomed with the same protein extracts as in **Figures 2** and **3**. (**b- next page**) Differentially expressed key genes in NER at mRNA level and their functional localisation.

**Figure S5.** **Representative autoradiograms for the Southern blot analysis of monohydroxymelphalan induced monoadducts in OFF and induced Saos2- and Li-Fraumeni-p21^WAF1/Cip1^ Tet-ON cells, respectively, (see Fig. 3b).**

**Figure S6.** **Models depicting proposed function of synthesis-dependent strand annealing (SDSA), break-induced replication (BIR) and single-strand annealing (SSA) repair mechanisms.** (**aii**) In the SDSA model, double-stranded breaks (DSB) are repaired by homologous recombination, without formation of a double Holliday junction, producing non-crossover products. In this model, following DSB recognition (MRN complex: MRE11/RAD50/NBS1) the DSB ends are recessed (by CtIP and EXO I, facilitated by SLX1/SLX4). The generated single-stranded 3' overhangs are then coated with the RPA protein, while RAD51 loading is facilitated (BRCA2/PALB2/DSS1). Subsequently, strand invasion by Rad51 single-stranded DNA nucleoprotein filaments takes place, forming a D-loop. The invading 3’ strand extends along the recipient homologous DNA duplex by DNA polymerase (Pol δ) in a 5’ to 3’ direction. As a result the D-loop “moves” in this process, which is termed as bubble migration DNA synthesis. The single Holliday junction also slides down the DNA duplex in the same direction in a process called branch migration, displacing the extended strand from the template strand. The newly synthesized 3' end of the invading strand is then able to anneal to the other 3' overhang in the damaged chromosome through complementary base pairing. After the strands anneal, a small flap of DNA can sometimes remain. Such flaps are removed, and the process finishes with the resealing, also known as ligation (LIG), of any remaining single-stranded gaps. [**51, 57, 58, 78-80**]

(**aii**) The break-induced replication (BIR) pathway is a homology-dependent repair route of one sided DSBs. During DNA replication, DSBs arising from stalled or collapse replication fork as well as DSBs emerging from erosion of uncapped telomeres are one end sided. They are considered to be repaired by strand invasion into a homologous duplex DNA followed by replication to the chromosome end (conservative replication). Specifically, the DNA at the DSB end is 5’ to 3’ recessed (by CtIP and EXO I facilitated by SLX1/SLX4), generating single-stranded 3' overhangs that are coated with the RPA protein, while RAD51 loading is facilitated (BRCA2/PALB2/DSS1). Subsequently, strand invasion by Rad51 single-stranded DNA nucleoprotein filaments takes place, forming a D-loop. The D-loop is not dissolved but moves together with the replication fork (migrating bubble - arrow showing direction of movement). The D-loop progresses with concomitant leading and lagging strand DNA synthesis (Pol D3). As lagging strand DNA synthesis delays, long single strand stretches (red arrow denoted) are produced. Following strand displacement gaps are filed and ligation (LIG) restores continuity of the DNA strand. The BIR pathway is considered mutagenic since it results in loss of heterozygosity or in a nonreciprocal translocation, if an ectopically template is employed. [**51, 57, 58, 78-80**]

(**b**) The single-strand annealing (SSA) pathway is a homology-dependent repair route of DSBs occurring between two repeat sequences. In contrast to the SDSA pathway, the SSA mechanism does not rely on the use of a separate homologous DNA strand for recombination. Instead, the SSA route requires only a single DNA duplex, using the repeat sequences flanking the DSB, as the homologous recombination sequences needed for repair. The steps of the SSA pathway are as follows. Initially, upon damage recognition (MRN complex: MRE11/RAD50/NBS1) the DNA around the DSB is recessed (CtIP and EXO I facilitated by SLX1/SLX4), generating single-stranded 3' overhangs that are coated with the RPA protein, thus preventing these overhangs from sticking to themselves. Subsequently, Rad52 binds the repeat sequences on each side of the break, and aligns them to enable the two complementary repeat sequences to anneal. Subsequently, the 3' non-homologous flaps are excised by the XPF/ERCC1 nucleases (denoted by black arrows), whose recruitment is facilitated by MSH2/MSH3. Gaps are filed and ligation (LIG) restores continuity of the DNA strands. The SSA pathway is considered mutagenic since the DNA sequence between the repeats and one of the two repeats are lost. [**51, 57, 58, 78-80**]

**Figure S7. Increased Rad52 expression upon sustained p21^WAF1/Cip1^ expression.** Quantitative analysis of Rad52 IF signal intensity in Saos2-p21^WAF1/Cip1^ Tet-ON cells, showing the high levels of Rad52 expression upon p21^WAF1/Cip1^ induction. A.U.: arbitrary units.

**Figure S8.** **E2F1 mediates *Rad52* transcriptional up-regulation upon extended p21^WAF1/Cip1^ expression.** Subchromosomal localization, gene organization and promoter sequence of the human *Rad52* locus. Analysis of the *Rad52* gene promoter (retrieved from Ensembl) using the online bioinformatic tool “GPMiner” (<http://gpminer.mbc.nctu.edu.tw/index.php>) that uses the Transfac database of mammalian transcriptional regulatory elements, revealed several putative transcription binding sites including E2F1.
