# Supplementary material for: Mutational signatures reveal the role of RAD52 in p53-independent p21-driven genomic instability
Source: Genome Biol. 2018 Mar 16;19:37. doi: 10.1186/s13059-018-1401-9 (PMC5857109; doi:10.1186/s13059-018-1401-9)
Supplement: Supplementary file 10 — Tables S1. to S4. (DOCX 71 kb) [file 13059_2018_1401_MOESM6_ESM.docx]

**ADDITIONAL FILE 2: Tables S1 to S4**

**Mutational signatures reveal the role of RAD52 in p53-independent p21 driven genomic instability**

Panagiotis Galanos^1,2*^, George Pappas^1,2*^, Alexander Polyzos^3^, Athanassios Kotsinas^1^, Ioanna Svolaki^1^, Nickolaos N Giakoumakis^4^, Christina Glytsou^5^, Ioannis S Pateras^1^, Umakanta Swain^6^, Vassilis L Souliotis^7^, Alexandros G Georgakilas^8^, Nicholas Geacintov^9^, Luca Scorrano^5^, Claudia Lukas^10^, Jiri Lukas^10^, Zvi Livneh^6^, Zoi Lygerou^4^, Dipanjan Chowdhury^11,12^, Claus Storgaard Sørensen^13^, Jiri Bartek^2,14**^, Vassilis G. Gorgoulis^1,3,15**^

* Authors equally contributed

** To whom correspondence should be addressed:

**Vassilis G. Gorgoulis,** E-mail: vgorg@med.uoa.gr; or: vgorgoulis@gmail.com;

Tel.: 0030-2107462352 **and** **Jiri Bartek**, E-mail: [jb@cancer.dk](mailto:jb@cancer.dk); Tel.: +45 – 35257357

**This file includes:**

Tables S1 to S4

**Suppl Table 1. DNA sequence analysis of TLS events across a site-specific BP-G (benzo[a]pyrene-guanine) adduct in the gapped plasmid TLS assay.**

| **Event type** | **Sequence opposite**  **BP-G^1^** | **Number of events (%)** | |
| --- | --- | --- | --- |
|  |  | **- DOX** | **+DOX** |
| Accurate TLS^2^ | 5’-TCA | 56 (59%) | 65 (71%) |
| Mutagenic TLS |  | **33 (35%)** | **9 (10%)** |
| Targeted^3^ | 5’-T***A***A | 23 | 3 |
|  | 5’-T***T***A | 2 | 2 |
|  | 5’-T***G***A | 6 | 2 |
| Untargeted^4^ |  | 2 | 2 |
| Non-TLS events |  | 6 (6%) | 18 (19%) |
| Total events |  | 95 (100%) | 92 (100%) |

^1^ The nucleotide inserted opposite the BP-G is underlined. Also shown are the 2 flanking nucleotides. Mutated nucleotides are in bold and Italic type.

^2^ TLS events in which the correct C were inserted opposite the BP-G.

^3^ TLS events in which a nucleotide other than C was inserted opposite the BP-G.

^4^ TLS events in which an incorrect nucleotide was inserted away from the lesion.

**Suppl Table 2a**. Chromosomal coordinates of breakpoints found by deep sequencing in Saos2 p21^WAF1/Cip1^ Tet-ON.

**1^st^ replicate** (152 novel breakpoints)

| Interacting Chromosome1 | Breakpoint CHR1 | # SNPs (+/-50kb from Breakpoint) | # INDELs (+/-50kb from Breakpoint) | Interacting Chromosome2 | Breakpoint CHR2 | # SNPs (+/-50kb from Breakpoint) | # INDELs (+/-50kb from Breakpoint) |
| --- | --- | --- | --- | --- | --- | --- | --- |
| chr1 | 22995595 | 9 | 0 | chr12 | 72261139 | 4 | 1 |
| chr1 | 29659277 | 2 | 1 | chr12 | 74411193 | 8 | 1 |
| chr1 | 41878753 | 0 | 0 | chr6 | 155498660 | 7 | 2 |
| chr1 | 58953316 | 2 | 2 | chr3 | 127910772 | 2 | 2 |
| chr1 | 58953316 | 2 | 2 | chr3 | 177557284 | 13 | 2 |
| chr1 | 86398983 | 1 | 0 | chr9 | 100675323 | 0 | 0 |
| chr1 | 108429135 | 11 | 2 | chr13 | 45444684 | 12 | 3 |
| chr1 | 145139441 | 48 | 5 | chr12 | 93771987 | 0 | 0 |
| chr1 | 164240570 | 0 | 0 | chr2 | 190999114 | 4 | 0 |
| chr10 | 74767644 | 0 | 0 | chr17 | 32673339 | 0 | 0 |
| chr14 | 37769600 | 1 | 0 | chr16 | 61079187 | 0 | 0 |
| chr16 | 33974188 | 108 | 11 | chr21 | 10701701 | 45 | 8 |
| chr16 | 54335710 | 0 | 0 | chr17 | 53683002 | 2 | 0 |
| chr16 | 58853574 | 2 | 0 | chrX | 139876054 | 3 | 1 |
| chr16 | 90064568 | 2 | 1 | chrX | 145688638 | 2 | 1 |
| chr19 | 31803235 | 1 | 0 | chr20 | 13232736 | 1 | 0 |
| chr19 | 48922102 | 2 | 0 | chr20 | 20964307 | 1 | 1 |
| chr2 | 81425909 | 12 | 0 | chr19 | 2723655 | 0 | 0 |
| chr2 | 92269899 | 9 | 0 | chr10 | 42599095 | 0 | 0 |
| chr2 | 151497250 | 0 | 0 | chr14 | 62337711 | 1 | 0 |
| chr2 | 153997824 | 0 | 0 | chr17 | 74377266 | 10 | 1 |
| chr2 | 176713737 | 1 | 0 | chr10 | 658147 | 0 | 0 |
| chr2 | 235906452 | 0 | 0 | chr13 | 54447843 | 3 | 0 |
| chr2 | 235906452 | 0 | 0 | chr20 | 24586704 | 4 | 0 |
| chr3 | 49441838 | 0 | 0 | chr12 | 86120300 | 0 | 0 |
| chr3 | 56468478 | 0 | 0 | chr4 | 15660385 | 2 | 0 |
| chr3 | 87637413 | 0 | 0 | chr12 | 8696300 | 3 | 0 |
| chr3 | 132109289 | 16 | 3 | chr14 | 31087222 | 3 | 2 |
| chr3 | 136061823 | 3 | 0 | chr12 | 34701446 | 1 | 1 |
| chr3 | 189767744 | 18 | 2 | chr6 | 31031442 | 3 | 2 |
| chr3 | 196141336 | 4 | 0 | chr5 | 158837608 | 1 | 0 |
| chr4 | 7735256 | 2 | 0 | chr8 | 135770882 | 17 | 3 |
| chr4 | 67662743 | 10 | 1 | chr8 | 114212420 | 17 | 1 |
| chr4 | 95518674 | 1 | 0 | chr7 | 46904550 | 0 | 1 |
| chr4 | 107184909 | 0 | 0 | chr20 | 34469478 | 1 | 0 |
| chr5 | 115449341 | 0 | 0 | chr7 | 111232192 | 6 | 0 |
| chr6 | 24684281 | 9 | 3 | chr22 | 32927887 | 1 | 0 |
| chr6 | 66251220 | 20 | 3 | chr17 | 50119287 | 3 | 1 |
| chr6 | 66594983 | 59 | 11 | chr16 | 54315636 | 0 | 0 |
| chr6 | 70863617 | 8 | 4 | chr7 | 120623106 | 2 | 0 |
| chr6 | 107308011 | 0 | 0 | chr8 | 105231721 | 0 | 0 |
| chr6 | 113723012 | 9 | 0 | chr14 | 25434286 | 3 | 0 |
| chr6 | 123171308 | 11 | 1 | chr15 | 70839461 | 0 | 0 |
| chr6 | 155237916 | 15 | 1 | chr8 | 33358442 | 0 | 0 |
| chr7 | 4350102 | 15 | 0 | chr8 | 137520123 | 4 | 1 |
| chr7 | 8744337 | 27 | 2 | chr15 | 83732109 | 2 | 1 |
| chr7 | 10349475 | 23 | 1 | chr21 | 17045052 | 2 | 0 |
| chr7 | 10349475 | 23 | 1 | chr8 | 138604161 | 5 | 0 |
| chr7 | 12546911 | 22 | 2 | chr8 | 106734493 | 5 | 1 |
| chr7 | 61794580 | 15 | 1 | chr16 | 46423423 | 13 | 0 |
| chr7 | 69163499 | 3 | 0 | chr20 | 1024652 | 1 | 0 |
| chr7 | 85878301 | 3 | 0 | chr20 | 1530547 | 0 | 0 |
| chr7 | 111053680 | 6 | 0 | chr12 | 108203004 | 0 | 0 |
| chr7 | 127601773 | 5 | 0 | chr9 | 257473 | 9 | 0 |
| chr8 | 15289683 | 0 | 0 | chr13 | 74313853 | 1 | 0 |
| chr8 | 23942911 | 0 | 0 | chr16 | 53553689 | 2 | 1 |
| chr8 | 30145553 | 0 | 0 | chr17 | 7167637 | 0 | 0 |
| chr8 | 96265942 | 0 | 0 | chr19 | 18835351 | 0 | 0 |
| chr8 | 107295989 | 25 | 6 | chr15 | 82137352 | 10 | 4 |
| chr8 | 107471437 | 4 | 3 | chr15 | 98589152 | 7 | 1 |
| chr8 | 107791903 | 15 | 2 | chr15 | 81846370 | 13 | 0 |
| chr8 | 134972379 | 18 | 3 | chr18 | 57070971 | 0 | 0 |
| chr8 | 137343657 | 11 | 2 | chr15 | 98378150 | 2 | 0 |
| chr8 | 142456681 | 2 | 0 | chrX | 141285536 | 0 | 0 |
| chr8 | 143459475 | 2 | 0 | chr17 | 56702967 | 4 | 2 |
| chr9 | 80932421 | 4 | 0 | chr12 | 2858529 | 0 | 0 |
| chr9 | 84660548 | 2 | 0 | chrX | 143759641 | 15 | 3 |
| chr9 | 88670487 | 0 | 0 | chrX | 142753696 | 2 | 0 |
| chr9 | 138976918 | 0 | 0 | chrX | 143708385 | 13 | 4 |
| chr9 | 139282240 | 0 | 0 | chr16 | 61294157 | 0 | 0 |
| chr9 | 139435562 | 1 | 0 | chr19 | 34937120 | 28 | 3 |
| chr1 | 25609873 | 0 | 0 | chr12 | 75898481 | 10 | 0 |
| chr1 | 35305148 | 1 | 0 | chr8 | 70364269 | 0 | 0 |
| chr1 | 75557407 | 14 | 3 | chr8 | 92083688 | 0 | 0 |
| chr1 | 83202568 | 5 | 4 | chrX | 10501384 | 0 | 0 |
| chr1 | 92116343 | 19 | 4 | chr10 | 35046048 | 0 | 0 |
| chr1 | 109495102 | 4 | 1 | chr3 | 110413224 | 0 | 0 |
| chr1 | 119401105 | 0 | 0 | chr15 | 51189033 | 0 | 0 |
| chr1 | 145465596 | 16 | 6 | chr14 | 59345299 | 0 | 0 |
| chr1 | 157732969 | 0 | 0 | chr13 | 61220984 | 3 | 1 |
| chr1 | 168186317 | 0 | 0 | chr3 | 53175880 | 0 | 0 |
| chr10 | 27775816 | 0 | 0 | chr12 | 11191225 | 90 | 5 |
| chr11 | 17215467 | 0 | 0 | chr19 | 32813560 | 0 | 0 |
| chr11 | 49883772 | 0 | 0 | chr17 | 22020663 | 0 | 0 |
| chr11 | 112779705 | 1 | 0 | chr20 | 58584350 | 6 | 1 |
| chr12 | 15199487 | 3 | 0 | chr17 | 72298101 | 5 | 3 |
| chr12 | 56990016 | 0 | 0 | chr15 | 39994414 | 0 | 0 |
| chr12 | 58469876 | 8 | 2 | chrX | 76361885 | 0 | 0 |
| chr13 | 39499757 | 0 | 0 | chr22 | 48035001 | 0 | 0 |
| chr13 | 63621213 | 121 | 3 | chr17 | 21666716 | 0 | 0 |
| chr14 | 19010480 | 109 | 0 | chr21 | 14339330 | 148 | 9 |
| chr15 | 65329146 | 0 | 0 | chr17 | 78055736 | 24 | 8 |
| chr15 | 72995309 | 0 | 0 | chr18 | 6521454 | 0 | 0 |
| chr16 | 33919702 | 6 | 3 | chr21 | 10709339 | 91 | 12 |
| chr16 | 33988260 | 108 | 11 | chr21 | 10714840 | 108 | 16 |
| chr16 | 46395482 | 4 | 0 | chr17 | 53572043 | 9 | 0 |
| chr16 | 46422897 | 13 | 0 | chr17 | 53683002 | 2 | 0 |
| chr16 | 46432310 | 13 | 0 | chr17 | 53562484 | 9 | 0 |
| chr16 | 46436312 | 13 | 0 | chr17 | 59589932 | 3 | 3 |
| chr17 | 54339339 | 6 | 0 | chrX | 141293787 | 0 | 0 |
| chr18 | 15405854 | 43 | 4 | chr21 | 14358785 | 148 | 9 |
| chr2 | 68914631 | 3 | 0 | chr5 | 64467875 | 0 | 0 |
| chr2 | 76593395 | 3 | 1 | chr4 | 162704554 | 1 | 0 |
| chr2 | 92269153 | 9 | 0 | chr7 | 61054333 | 4 | 0 |
| chr2 | 92269899 | 9 | 0 | chr10 | 42390282 | 11 | 11 |
| chr2 | 92269899 | 9 | 0 | chr10 | 42394735 | 11 | 11 |
| chr2 | 92269899 | 9 | 0 | chr10 | 42596687 | 0 | 0 |
| chr2 | 92316241 | 4 | 0 | chr18 | 18520043 | 0 | 0 |
| chr2 | 115834058 | 0 | 0 | chr19 | 21686845 | 1 | 0 |
| chr2 | 132987659 | 96 | 5 | chr15 | 20000339 | 64 | 3 |
| chr2 | 165406365 | 0 | 0 | chr9 | 38303914 | 1 | 0 |
| chr2 | 177316676 | 1 | 0 | chr7 | 133023275 | 3 | 0 |
| chr2 | 185918365 | 0 | 0 | chr8 | 125889664 | 2 | 1 |
| chr2 | 236990161 | 0 | 0 | chr4 | 180114228 | 0 | 0 |
| chr3 | 49218288 | 0 | 0 | chrX | 32000795 | 0 | 0 |
| chr3 | 49441767 | 0 | 0 | chr10 | 17590352 | 0 | 0 |
| chr3 | 50879412 | 0 | 0 | chr5 | 81987224 | 0 | 0 |
| chr3 | 109751802 | 0 | 0 | chr6 | 78649478 | 6 | 1 |
| chr3 | 137221719 | 6 | 1 | chr9 | 2396941 | 0 | 0 |
| chr3 | 183430104 | 18 | 3 | chr10 | 96291400 | 0 | 0 |
| chr3 | 189767744 | 18 | 2 | chr9 | 118356960 | 1 | 0 |
| chr4 | 146916 | 4 | 2 | chr10 | 101851919 | 0 | 0 |
| chr4 | 13785317 | 7 | 0 | chr20 | 29641618 | 8 | 1 |
| chr4 | 74243784 | 0 | 0 | chr5 | 39794023 | 14 | 1 |
| chr4 | 79750923 | 0 | 0 | chr20 | 49282441 | 4 | 0 |
| chr4 | 79750923 | 0 | 0 | chr6 | 30820247 | 21 | 3 |
| chr4 | 154401330 | 0 | 1 | chr17 | 70856088 | 37 | 11 |
| chr5 | 36978437 | 9 | 0 | chrX | 74159762 | 1 | 0 |
| chr5 | 49405882 | 0 | 0 | chr19 | 24619912 | 22 | 1 |
| chr5 | 54866069 | 0 | 0 | chr10 | 75489047 | 0 | 0 |
| chr5 | 84457596 | 0 | 0 | chr19 | 46262140 | 0 | 0 |
| chr6 | 13718797 | 3 | 0 | chr12 | 107337314 | 0 | 0 |
| chr6 | 26446989 | 24 | 3 | chrX | 85312990 | 0 | 0 |
| chr6 | 57403740 | 480 | 52 | chr8 | 41279542 | 10 | 0 |
| chr6 | 68130643 | 22 | 2 | chr16 | 46427287 | 13 | 0 |
| chr6 | 71180868 | 1 | 0 | chr16 | 46423423 | 13 | 0 |
| chr6 | 71181986 | 2 | 0 | chr17 | 59586616 | 3 | 3 |
| chr6 | 131057741 | 14 | 0 | chr9 | 94148208 | 0 | 0 |
| chr6 | 154216018 | 8 | 0 | chr21 | 9422801 | 0 | 0 |
| chr6 | 158908435 | 4 | 2 | chr12 | 117819161 | 0 | 0 |
| chr7 | 5120579 | 4 | 2 | chr8 | 133881743 | 6 | 0 |
| chr7 | 5896162 | 1 | 0 | chr20 | 35627761 | 0 | 0 |
| chr7 | 27804657 | 0 | 0 | chr12 | 63946476 | 5 | 1 |
| chr7 | 33195542 | 0 | 0 | chr19 | 53239920 | 1 | 0 |
| chr7 | 61754908 | 17 | 1 | chr16 | 46441980 | 13 | 0 |
| chr7 | 86154768 | 1 | 0 | chr18 | 49931825 | 0 | 0 |
| chr7 | 86640766 | 4 | 2 | chr11 | 93487901 | 0 | 0 |
| chr7 | 101031692 | 2 | 0 | chr17 | 787108 | 0 | 0 |
| chr8 | 52731369 | 2 | 0 | chr11 | 38812433 | 0 | 0 |
| chr8 | 137519407 | 4 | 1 | chr21 | 17031589 | 2 | 0 |
| chr9 | 69957723 | 4 | 1 | chr18 | 15405704 | 43 | 4 |
| chr9 | 132186820 | 13 | 1 | chr22 | 23852643 | 1 | 0 |

**2^nd^ replicate** (176 breakpoints)

| Interacting Chromosome1 | Breakpoint CHR1 | # SNPs (+/-50kb from Breakpoint) | # INDELs (+/-50kb from Breakpoint) | Interacting Chromosome2 | Breakpoint CHR2 | # SNPs (+/-50kb from Breakpoint) | # INDELs (+/-50kb from Breakpoint) |
| --- | --- | --- | --- | --- | --- | --- | --- |
| chr1 | 22995595 | 6 | 3 | chr12 | 72261139 | 0 | 1 |
| chr1 | 29659277 | 0 | 0 | chr12 | 74411193 | 1 | 0 |
| chr1 | 41878753 | 0 | 0 | chr6 | 155498660 | 0 | 1 |
| chr1 | 58953316 | 1 | 0 | chr3 | 127910772 | 1 | 0 |
| chr1 | 58953316 | 1 | 0 | chr3 | 177557284 | 6 | 1 |
| chr1 | 86398983 | 0 | 1 | chr9 | 100675323 | 0 | 0 |
| chr1 | 108429135 | 0 | 0 | chr13 | 45444684 | 1 | 0 |
| chr1 | 145139441 | 13 | 4 | chr12 | 93771987 | 0 | 0 |
| chr1 | 164240570 | 0 | 0 | chr2 | 190999114 | 5 | 0 |
| chr10 | 74767644 | 0 | 0 | chr17 | 32673339 | 0 | 0 |
| chr14 | 37769600 | 0 | 0 | chr16 | 61079187 | 0 | 0 |
| chr16 | 33974188 | 72 | 11 | chr21 | 10701701 | 42 | 11 |
| chr16 | 54335710 | 0 | 0 | chr17 | 53683002 | 0 | 1 |
| chr16 | 58853574 | 1 | 0 | chrX | 139876054 | 3 | 0 |
| chr16 | 90064568 | 3 | 1 | chrX | 145688638 | 2 | 1 |
| chr19 | 31803235 | 1 | 1 | chr20 | 13232736 | 0 | 0 |
| chr19 | 48922102 | 0 | 0 | chr20 | 20964307 | 1 | 1 |
| chr2 | 81425909 | 0 | 0 | chr19 | 2723655 | 0 | 1 |
| chr2 | 92269899 | 5 | 1 | chr10 | 42599095 | 5 | 0 |
| chr2 | 151497250 | 0 | 0 | chr14 | 62337711 | 0 | 0 |
| chr2 | 153997824 | 0 | 0 | chr17 | 74377266 | 1 | 3 |
| chr2 | 176713737 | 0 | 0 | chr10 | 658147 | 0 | 0 |
| chr2 | 235906452 | 0 | 0 | chr13 | 54447843 | 0 | 0 |
| chr2 | 235906452 | 0 | 0 | chr20 | 24586704 | 0 | 0 |
| chr3 | 49441838 | 0 | 0 | chr12 | 86120300 | 0 | 0 |
| chr3 | 56468478 | 0 | 0 | chr4 | 15660385 | 0 | 0 |
| chr3 | 87637413 | 0 | 0 | chr12 | 8696300 | 0 | 1 |
| chr3 | 132109289 | 4 | 0 | chr14 | 31087222 | 0 | 0 |
| chr3 | 136061823 | 0 | 0 | chr12 | 34701446 | 2 | 0 |
| chr3 | 189767744 | 2 | 0 | chr6 | 31031442 | 8 | 0 |
| chr3 | 196141336 | 2 | 0 | chr5 | 158837608 | 0 | 0 |
| chr4 | 7735256 | 4 | 0 | chr8 | 135770882 | 1 | 0 |
| chr4 | 67662743 | 1 | 0 | chr8 | 114212420 | 1 | 1 |
| chr4 | 95518674 | 0 | 0 | chr7 | 46904550 | 0 | 0 |
| chr4 | 107184909 | 0 | 0 | chr20 | 34469478 | 0 | 0 |
| chr5 | 115449341 | 0 | 0 | chr7 | 111232192 | 1 | 1 |
| chr6 | 24684281 | 3 | 0 | chr22 | 32927887 | 0 | 0 |
| chr6 | 66251220 | 0 | 0 | chr17 | 50119287 | 1 | 1 |
| chr6 | 66594983 | 4 | 0 | chr16 | 54315636 | 0 | 1 |
| chr6 | 70863617 | 0 | 1 | chr7 | 120623106 | 0 | 0 |
| chr6 | 107308011 | 5 | 0 | chr8 | 105231721 | 0 | 0 |
| chr6 | 113723012 | 2 | 0 | chr14 | 25434286 | 1 | 1 |
| chr6 | 123171308 | 3 | 0 | chr15 | 70839461 | 0 | 0 |
| chr6 | 155237916 | 0 | 3 | chr8 | 33358442 | 0 | 0 |
| chr7 | 4350102 | 2 | 2 | chr8 | 137520123 | 1 | 0 |
| chr7 | 8744337 | 11 | 1 | chr15 | 83732109 | 1 | 0 |
| chr7 | 10349475 | 6 | 1 | chr21 | 17045052 | 1 | 1 |
| chr7 | 10349475 | 6 | 1 | chr8 | 138604161 | 2 | 1 |
| chr7 | 12546911 | 1 | 0 | chr8 | 106734493 | 1 | 2 |
| chr7 | 61794580 | 21 | 1 | chr16 | 46423423 | 16 | 0 |
| chr7 | 69163499 | 0 | 0 | chr20 | 1024652 | 0 | 0 |
| chr7 | 85878301 | 0 | 0 | chr20 | 1530547 | 0 | 0 |
| chr7 | 111053680 | 2 | 0 | chr12 | 108203004 | 0 | 0 |
| chr7 | 127601773 | 2 | 1 | chr9 | 257473 | 4 | 0 |
| chr8 | 15289683 | 0 | 0 | chr13 | 74313853 | 0 | 1 |
| chr8 | 23942911 | 0 | 0 | chr16 | 53553689 | 0 | 0 |
| chr8 | 30145553 | 0 | 0 | chr17 | 7167637 | 0 | 0 |
| chr8 | 96265942 | 0 | 0 | chr19 | 18835351 | 4 | 0 |
| chr8 | 107295989 | 0 | 6 | chr15 | 82137352 | 4 | 4 |
| chr8 | 107471437 | 0 | 0 | chr15 | 98589152 | 3 | 5 |
| chr8 | 107791903 | 2 | 1 | chr15 | 81846370 | 3 | 0 |
| chr8 | 134972379 | 2 | 0 | chr18 | 57070971 | 0 | 0 |
| chr8 | 137343657 | 0 | 2 | chr15 | 98378150 | 1 | 1 |
| chr8 | 142456681 | 6 | 2 | chrX | 141285536 | 2 | 0 |
| chr8 | 143459475 | 3 | 1 | chr17 | 56702967 | 1 | 1 |
| chr9 | 80932421 | 1 | 2 | chr12 | 2858529 | 0 | 0 |
| chr9 | 84660548 | 0 | 0 | chrX | 143759641 | 3 | 1 |
| chr9 | 88670487 | 1 | 0 | chrX | 142753696 | 0 | 0 |
| chr9 | 138976918 | 2 | 0 | chrX | 143708385 | 2 | 1 |
| chr9 | 139282240 | 0 | 0 | chr16 | 61294157 | 0 | 1 |
| chr9 | 139435562 | 1 | 1 | chr19 | 34937120 | 12 | 6 |
| chr1 | 18875185 | 12 | 2 | chr6 | 169513119 | 1 | 1 |
| chr1 | 19168650 | 5 | 3 | chr2 | 107318330 | 0 | 1 |
| chr1 | 30542516 | 0 | 2 | chr6 | 66079740 | 0 | 2 |
| chr1 | 37932671 | 1 | 1 | chr12 | 76644094 | 0 | 0 |
| chr1 | 41878651 | 0 | 0 | chr3 | 180480797 | 5 | 0 |
| chr1 | 46983960 | 0 | 0 | chr6 | 170571502 | 0 | 0 |
| chr1 | 52372701 | 2 | 0 | chr15 | 100271274 | 9 | 3 |
| chr1 | 80618242 | 0 | 3 | chr21 | 37560755 | 1 | 0 |
| chr1 | 85760860 | 1 | 2 | chr7 | 133873236 | 0 | 0 |
| chr1 | 85988270 | 1 | 4 | chr12 | 48181894 | 0 | 0 |
| chr1 | 88931768 | 0 | 3 | chr3 | 181077346 | 4 | 0 |
| chr1 | 88972902 | 0 | 2 | chr17 | 32063849 | 0 | 0 |
| chr1 | 98986901 | 2 | 1 | chr3 | 173716247 | 1 | 0 |
| chr1 | 112363911 | 0 | 0 | chr14 | 52529972 | 0 | 1 |
| chr1 | 118448152 | 0 | 0 | chr10 | 114593481 | 0 | 0 |
| chr1 | 119401178 | 0 | 0 | chr2 | 141852714 | 0 | 0 |
| chr1 | 121485366 | 0 | 0 | chr19 | 27736394 | 1 | 0 |
| chr1 | 121485366 | 0 | 0 | chr19 | 58047324 | 3 | 0 |
| chr1 | 121485366 | 0 | 0 | chr19 | 58876598 | 0 | 0 |
| chr1 | 142539679 | 43 | 5 | chr21 | 10084924 | 0 | 0 |
| chr1 | 145585416 | 0 | 5 | chr11 | 70220497 | 0 | 0 |
| chr1 | 146754100 | 3 | 4 | chr16 | 62663221 | 1 | 0 |
| chr1 | 159906922 | 0 | 1 | chr10 | 50686304 | 0 | 0 |
| chr1 | 178664936 | 0 | 1 | chr5 | 173541367 | 5 | 5 |
| chr1 | 215043885 | 0 | 0 | chr9 | 38241123 | 0 | 0 |
| chr10 | 17590511 | 0 | 0 | chr12 | 86120303 | 0 | 0 |
| chr10 | 133879438 | 0 | 1 | chr20 | 42425523 | 1 | 0 |
| chr11 | 18400230 | 0 | 0 | chr13 | 28649669 | 0 | 0 |
| chr11 | 24762009 | 0 | 0 | chr21 | 44356238 | 30 | 1 |
| chr11 | 37429138 | 0 | 0 | chr17 | 10138572 | 0 | 0 |
| chr12 | 4526554 | 0 | 0 | chr13 | 45479288 | 1 | 0 |
| chr12 | 6921609 | 1 | 0 | chr19 | 34022618 | 18 | 1 |
| chr12 | 15199579 | 2 | 0 | chr19 | 57649072 | 1 | 0 |
| chr12 | 29243974 | 2 | 0 | chr19 | 33709262 | 17 | 5 |
| chr13 | 48588992 | 0 | 0 | chr17 | 70381740 | 2 | 2 |
| chr13 | 51176932 | 0 | 0 | chr21 | 9826850 | 33 | 1 |
| chr13 | 60843705 | 3 | 1 | chr18 | 72941720 | 0 | 0 |
| chr13 | 63637800 | 71 | 2 | chr20 | 26148033 | 0 | 1 |
| chr13 | 105798436 | 0 | 0 | chr15 | 90328124 | 0 | 0 |
| chr13 | 113455291 | 1 | 1 | chr16 | 3725933 | 0 | 0 |
| chr14 | 47880853 | 1 | 0 | chr19 | 35762946 | 1 | 2 |
| chr14 | 86020457 | 0 | 0 | chr20 | 14960879 | 0 | 0 |
| chr15 | 38996443 | 0 | 0 | chr21 | 42832513 | 4 | 0 |
| chr16 | 46410529 | 16 | 0 | chr17 | 53687466 | 0 | 1 |
| chr17 | 70375326 | 2 | 2 | chr18 | 46312719 | 0 | 0 |
| chr19 | 34233283 | 32 | 4 | chr20 | 20250761 | 2 | 1 |
| chr2 | 2702709 | 3 | 0 | chr6 | 119541034 | 1 | 2 |
| chr2 | 3790147 | 3 | 0 | chr4 | 30551155 | 0 | 0 |
| chr2 | 27527648 | 0 | 1 | chr3 | 194926826 | 2 | 0 |
| chr2 | 42052553 | 2 | 0 | chr4 | 66413651 | 0 | 0 |
| chr2 | 50675178 | 6 | 0 | chr3 | 185065176 | 13 | 0 |
| chr2 | 71436334 | 1 | 1 | chr12 | 91535030 | 0 | 0 |
| chr2 | 86025154 | 1 | 1 | chr11 | 66647844 | 0 | 0 |
| chr2 | 92269645 | 5 | 1 | chr3 | 196625613 | 2 | 0 |
| chr2 | 99501407 | 0 | 0 | chr5 | 68568047 | 0 | 0 |
| chr2 | 114228513 | 0 | 0 | chr6 | 39149897 | 0 | 0 |
| chr2 | 137168402 | 0 | 0 | chr9 | 18850445 | 0 | 0 |
| chr20 | 11281800 | 0 | 0 | chrX | 81651271 | 0 | 0 |
| chr21 | 30088615 | 0 | 0 | chrX | 83883241 | 0 | 0 |
| chr3 | 9923725 | 0 | 0 | chr12 | 99136388 | 0 | 0 |
| chr3 | 29817947 | 0 | 0 | chr8 | 131334445 | 0 | 0 |
| chr3 | 36540064 | 0 | 0 | chr13 | 55767092 | 0 | 0 |
| chr3 | 135176573 | 4 | 2 | chr5 | 66191957 | 0 | 0 |
| chr3 | 156835280 | 1 | 0 | chr6 | 84285548 | 2 | 0 |
| chr3 | 172749057 | 1 | 1 | chr5 | 160435506 | 0 | 0 |
| chr3 | 176465710 | 4 | 0 | chr10 | 72546708 | 0 | 1 |
| chr3 | 196625703 | 2 | 0 | chr10 | 42384962 | 14 | 8 |
| chr3 | 196625703 | 2 | 0 | chr10 | 42394850 | 14 | 8 |
| chr3 | 196625703 | 2 | 0 | chr10 | 42396571 | 14 | 8 |
| chr3 | 196625703 | 2 | 0 | chr10 | 42596689 | 5 | 0 |
| chr3 | 196625703 | 2 | 0 | chr10 | 42598316 | 5 | 0 |
| chr4 | 81311687 | 0 | 0 | chr18 | 73952001 | 0 | 0 |
| chr4 | 170281154 | 1 | 0 | chr14 | 52667543 | 17 | 0 |
| chr4 | 188901723 | 1 | 0 | chr9 | 94212211 | 0 | 0 |
| chr5 | 9064124 | 0 | 0 | chr6 | 129451605 | 0 | 0 |
| chr5 | 21573421 | 2 | 1 | chr6 | 57575758 | 191 | 11 |
| chr5 | 41428721 | 0 | 0 | chr8 | 111632283 | 0 | 0 |
| chr5 | 83790582 | 0 | 0 | chr8 | 133950312 | 9 | 4 |
| chr5 | 159349755 | 3 | 0 | chrX | 66982630 | 1 | 1 |
| chr5 | 171932228 | 1 | 3 | chr6 | 28864124 | 1 | 2 |
| chr5 | 173245820 | 6 | 3 | chr13 | 34928046 | 0 | 0 |
| chr6 | 25237447 | 1 | 1 | chr15 | 98630546 | 2 | 2 |
| chr6 | 30177779 | 1 | 0 | chr15 | 94947921 | 0 | 0 |
| chr6 | 31031991 | 8 | 0 | chr20 | 34469478 | 0 | 0 |
| chr6 | 66596731 | 3 | 0 | chr17 | 53690008 | 0 | 1 |
| chr7 | 13230695 | 0 | 0 | chr8 | 110416045 | 2 | 1 |
| chr7 | 16791645 | 0 | 0 | chr8 | 827458 | 0 | 0 |
| chr7 | 61796835 | 21 | 1 | chr16 | 46417216 | 16 | 0 |
| chr7 | 61891306 | 23 | 2 | chr16 | 32543194 | 96 | 15 |
| chr7 | 81789239 | 0 | 0 | chr10 | 60902430 | 0 | 0 |
| chr7 | 85284707 | 0 | 0 | chr9 | 100380621 | 0 | 0 |
| chr7 | 103455711 | 1 | 0 | chr8 | 118317997 | 1 | 3 |
| chr7 | 125264399 | 2 | 1 | chr12 | 74014370 | 3 | 0 |
| chr8 | 65122750 | 0 | 0 | chr9 | 138970121 | 2 | 0 |
| chr8 | 66883924 | 0 | 0 | chr17 | 53643786 | 2 | 3 |
| chr8 | 83459578 | 0 | 0 | chr15 | 81848807 | 3 | 0 |
| chr8 | 121534065 | 2 | 1 | chr11 | 48005298 | 0 | 0 |
| chr8 | 124964478 | 0 | 2 | chrX | 30712848 | 1 | 0 |
| chr8 | 131811374 | 1 | 0 | chr22 | 42630344 | 0 | 0 |
| chr8 | 136495867 | 4 | 5 | chr9 | 37363596 | 0 | 0 |
| chr8 | 138604234 | 2 | 1 | chr21 | 17045052 | 1 | 1 |
| chr9 | 11351815 | 0 | 0 | chr11 | 15698907 | 0 | 0 |
| chr9 | 79732630 | 0 | 0 | chr19 | 49554768 | 7 | 5 |
| chr9 | 140168709 | 0 | 1 | chr17 | 56779121 | 1 | 2 |

**Suppl Table 2b**. Chromosomal coordinates of breakpoints found by deep sequencing in Li-Fraumeni p21^WAF1/Cip1^ Tet-ON.

**1^st^ replicate** (44 novel breakpoints)

| Interacting Chromosome1 | Breakpoint CHR1 | # SNPs (+/-50kb from Breakpoint) | # INDELs (+/-50kb from Breakpoint) | Interacting Chromosome2 | Breakpoint CHR2 | # SNPs (+/-50kb from Breakpoint) | # INDELs (+/-50kb from Breakpoint) |
| --- | --- | --- | --- | --- | --- | --- | --- |
| chr13 | 19283307 | 1 | 0 | chr21 | 23199387 | 1 | 0 |
| chr5 | 993367 | 2 | 0 | chr9 | 23405883 | 0 | 0 |
| chr7 | 68251560 | 0 | 0 | chr8 | 82537218 | 8 | 0 |
| chr1 | 109495134 | 2 | 0 | chr3 | 110413098 | 2 | 0 |
| chr1 | 142655737 | 6 | 2 | chr21 | 23072353 | 1 | 5 |
| chr21 | 11022635 | 1 | 2 | chr22 | 45991513 | 3 | 0 |
| chr3 | 56766056 | 3 | 1 | chr7 | 147539088 | 1 | 0 |
| chr7 | 127930732 | 0 | 0 | chr16 | 76865434 | 0 | 0 |
| chr9 | 127885383 | 3 | 0 | chr18 | 71711145 | 0 | 0 |
| chr1 | 81817021 | 0 | 0 | chr7 | 12622723 | 0 | 0 |
| chr1 | 119401198 | 0 | 0 | chr2 | 141852622 | 0 | 0 |
| chr1 | 145139275 | 29 | 5 | chr12 | 93771974 | 1 | 0 |
| chr1 | 158991610 | 1 | 0 | chr6 | 160521749 | 0 | 0 |
| chr1 | 165706663 | 2 | 0 | chr12 | 32409422 | 1 | 0 |
| chr1 | 229733724 | 0 | 0 | chr21 | 20493380 | 0 | 0 |
| chr1 | 236941536 | 4 | 0 | chr5 | 100849578 | 0 | 1 |
| chr10 | 74767595 | 0 | 0 | chr17 | 32673526 | 0 | 0 |
| chr15 | 80893747 | 0 | 0 | chr22 | 34001222 | 0 | 0 |
| chr19 | 33444431 | 0 | 0 | chr20 | 26220470 | 3 | 1 |
| chr2 | 3931870 | 0 | 0 | chr12 | 124457691 | 1 | 0 |
| chr2 | 33041975 | 0 | 0 | chr11 | 87094468 | 0 | 0 |
| chr2 | 42052631 | 2 | 0 | chr4 | 66413934 | 0 | 0 |
| chr2 | 174048353 | 1 | 0 | chr10 | 116805541 | 0 | 0 |
| chr21 | 11151034 | 23 | 9 | chr22 | 17496293 | 17 | 1 |
| chr3 | 53902387 | 0 | 0 | chr5 | 32020538 | 4 | 0 |
| chr3 | 57578216 | 1 | 0 | chr21 | 23147151 | 0 | 2 |
| chr3 | 57843287 | 2 | 0 | chr18 | 75853501 | 2 | 0 |
| chr3 | 159504054 | 9 | 1 | chr13 | 62054094 | 0 | 0 |
| chr4 | 80894439 | 0 | 0 | chr12 | 9134433 | 5 | 0 |
| chr5 | 8582150 | 3 | 1 | chr7 | 83731611 | 0 | 0 |
| chr5 | 8915935 | 3 | 0 | chr17 | 81021800 | 0 | 0 |
| chr5 | 89451612 | 0 | 0 | chr10 | 124457729 | 1 | 0 |
| chr5 | 106533421 | 0 | 0 | chr8 | 126954041 | 20 | 1 |
| chr5 | 112881856 | 1 | 2 | chr13 | 46920678 | 0 | 0 |
| chr6 | 22882405 | 2 | 0 | chr14 | 83577782 | 0 | 0 |
| chr6 | 47324229 | 1 | 1 | chr13 | 105141245 | 0 | 0 |
| chr6 | 147605867 | 0 | 0 | chr11 | 106284502 | 0 | 0 |
| chr7 | 57444240 | 2 | 1 | chr20 | 48982875 | 9 | 0 |
| chr7 | 117228218 | 0 | 0 | chr15 | 75010518 | 4 | 0 |
| chr8 | 102173417 | 8 | 1 | chr11 | 10535957 | 2 | 1 |
| chr8 | 126611377 | 26 | 0 | chr15 | 81423347 | 1 | 0 |
| chr9 | 38243820 | 0 | 0 | chr15 | 63769764 | 0 | 0 |
| chr9 | 68339802 | 6 | 4 | chr21 | 9833903 | 22 | 2 |
| chr9 | 99999958 | 0 | 0 | chr13 | 41496784 | 0 | 0 |

**2^nd^ replicate** (34 breakpoints)

| Interacting Chromosome1 | Breakpoint CHR1 | # SNPs (+/-50kb from Breakpoint) | # INDELs (+/-50kb from Breakpoint) | Interacting Chromosome2 | Breakpoint CHR2 | # SNPs (+/-50kb from Breakpoint) | # INDELs (+/-50kb from Breakpoint) |
| --- | --- | --- | --- | --- | --- | --- | --- |
| chr13 | 19283307 | 1 | 0 | chr21 | 23199387 | 0 | 0 |
| chr5 | 993367 | 0 | 0 | chr9 | 23405883 | 0 | 1 |
| chr7 | 68251560 | 0 | 0 | chr8 | 82537218 | 2 | 0 |
| chr1 | 109495134 | 0 | 0 | chr3 | 110413098 | 0 | 0 |
| chr1 | 142655737 | 1 | 1 | chr21 | 23072353 | 2 | 6 |
| chr21 | 11022635 | 3 | 3 | chr22 | 45991513 | 1 | 0 |
| chr3 | 56766056 | 0 | 0 | chr7 | 147539088 | 0 | 0 |
| chr7 | 127930732 | 0 | 0 | chr16 | 76865434 | 0 | 0 |
| chr9 | 127885383 | 0 | 0 | chr18 | 71711145 | 0 | 0 |
| chr1 | 16517606 | 0 | 0 | chr5 | 24017642 | 1 | 1 |
| chr1 | 28515550 | 0 | 0 | chr19 | 24027601 | 1 | 0 |
| chr1 | 110122241 | 0 | 0 | chr14 | 107287328 | 1 | 0 |
| chr1 | 145112510 | 46 | 3 | chr6 | 136937085 | 0 | 0 |
| chr1 | 164240567 | 0 | 0 | chr2 | 190999060 | 0 | 0 |
| chr10 | 43492929 | 6 | 0 | chr17 | 42848142 | 0 | 0 |
| chr2 | 69840463 | 0 | 0 | chr7 | 140161339 | 0 | 0 |
| chr2 | 91937184 | 0 | 0 | chr12 | 123873263 | 0 | 0 |
| chr3 | 111274373 | 4 | 1 | chr8 | 128533541 | 21 | 0 |
| chr3 | 154257531 | 0 | 0 | chr7 | 6243936 | 1 | 0 |
| chr3 | 155750589 | 2 | 0 | chr8 | 120611734 | 4 | 1 |
| chr4 | 80894398 | 0 | 0 | chr12 | 9134451 | 1 | 0 |
| chr4 | 80894398 | 0 | 0 | chr5 | 21207740 | 3 | 2 |
| chr5 | 7899008 | 0 | 0 | chrX | 28106212 | 0 | 0 |
| chr5 | 21211111 | 4 | 2 | chr6 | 62563734 | 0 | 0 |
| chr5 | 30159436 | 2 | 1 | chr9 | 8685527 | 2 | 0 |
| chr5 | 31711991 | 1 | 0 | chr11 | 38369518 | 2 | 1 |
| chr5 | 37709727 | 0 | 0 | chr7 | 8663395 | 0 | 0 |
| chr5 | 159349909 | 0 | 0 | chrX | 66982563 | 0 | 0 |
| chr5 | 165001172 | 0 | 0 | chr10 | 18647312 | 4 | 1 |
| chr6 | 24684235 | 1 | 0 | chr22 | 32928333 | 0 | 0 |
| chr7 | 83721663 | 0 | 0 | chr8 | 130227165 | 5 | 0 |
| chr7 | 86339257 | 0 | 0 | chr8 | 79039465 | 2 | 0 |
| chr8 | 30145509 | 0 | 0 | chr17 | 7167717 | 0 | 0 |
| chr8 | 66057267 | 0 | 0 | chr10 | 105904570 | 0 | 0 |

**Suppl Table 3.** Primers and annealing temperatures used in real time RT-PCR and ChIP analyses.

| **Locus** | **Primers** | **Sequence** | **Application** | **Ann.** |
| --- | --- | --- | --- | --- |
|  |  |  |  | **Temp.(°C)** |
|  |  |  |  |  |
| *BRCA1* | Fw: | 5' - GGCTATCCTCTCAGAGTGACA - 3' | quantitative real-time RT-PCR | 58 |
| (set1) | Rv: | 5' - CTGATGTGCTTTGTTCTGGA - 3' |  |  |
|  |  |  |  |  |
| *BRCA1* | Fw: | 5' - GTCCCATCTGTCTGGAGTTGAT - 3' | quantitative real-time RT-PCR | 56 |
| (set 2) | Rv: | 5' - GGCCCTTTCTTCTGGTTGAGA - 3' |  |  |
|  |  |  |  |  |
| *BRCA1* | Fw: | 5' - GCTCTTCGCGTTGAAGT - 3' | quantitative real-time RT-PCR | 58 |
| (set 3) | Rv: | 5' - TCAACTCCAGACAGATGGGAC - 3' |  |  |
|  |  |  |  |  |
| *BRCA2* | Fw: | 5'- GAAGCGTGAGGGGACAGATT -3' | quantitative real-time RT-PCR | 57 |
| (set 1) | Rv: | 5'- AATAAGTCCGCTCCAGAGGTG -3' |  |  |
|  |  |  |  |  |
| *BRCA2* | Fw: | 5'- CTGTGTACCCTTYCGCACAAC -3' | quantitative real-time RT-PCR | 60 |
| (set 2) | Rv: | 5'- TCTTGACCAGGTGCGGTAAA -3' |  |  |
|  |  |  |  |  |
| *BRCA2* | Fw: | 5'- AAGCACTCCAGATGGCACAAT -3' | quantitative real-time RT-PCR | 58 |
| (set 3) | Rv: | 5'- GTGCGAAAGGGTACACAGGTA -3' |  |  |
|  |  |  |  |  |
| *RAD52* | Fw: | 5'- ATGCTTTGGACAGTGCCAGT - 3' | quantitative real-time RT-PCR | 60 |
| (set 1) | Rv: | 5'- CCAGCCATGCGGCTACTTAT -3' |  |  |
|  |  |  |  |  |
| *RAD52* | Fw: | 5'- CCCAGAACAGGACTCCACAC - 3' | quantitative real-time RT-PCR | 60 |
| (set 2) | Rv: | 5'- CCAGTTTCCTGTTGTGCGTT - 3' |  |  |
|  |  |  |  |  |
| *EXO1* | Fw: | 5’- GGCTTGCTAATCTTCCAGAGG -3’ | quantitative real-time RT-PCR | 60 |
|  | Rv: | 5’- CAGGCAAACTTCCCGAAATA -3’ |  |  |
|  |  |  |  |  |
| *APEX1* | Fw: | 5’- TGAAGCCTTTCGCAAGTTCCT -3’ | quantitative real-time RT-PCR | 62 |
|  | Rv: | 5’- TGAGGTCTCCACACAGCACAA -3’ |  |  |
|  |  |  |  |  |
| *MLH3* | Fw: | 5’- ccaaaccaatcgtccgtaag -3’ | quantitative real-time RT-PCR | 60 |
|  | Rv: | 5’- catcagagttctctaagcggttc -3’ |  |  |
|  |  |  |  |  |
| *DDB2* | Fw: | 5’- CCACCTTCATCAAAGGGATTGG -3’ | quantitative real-time RT-PCR | 58 |
|  | Rv: | 5’- CTCGGATCTCGCTCTTCTGGTC -3’ |  |  |
|  |  |  |  |  |
| *LIG3* | Fw: | 5’- GATCACGTGCCACCTACCTTGT -3’ | quantitative real-time RT-PCR | 66 |
|  | Rv: | 5’- GGCATAGTCCACACAGAACCGT- -3’ |  |  |
|  |  |  |  |  |
| *XPC* | Fw: | 5’- AAGACTTGGAGTTTCAGGCAAAA -3’ | quantitative real-time RT-PCR | 58 |
|  | Rv: | 5’- TCAGGAGATGCCGCTTCAG -3’ |  |  |
|  |  |  |  |  |
| *RAD23B* | Fw: | 5’- TTTACGGAATCAGCCTCAGTTT -3’ | quantitative real-time RT-PCR | 58 |
|  | Rv: | 5’- TGAATAAAATGCTCCTGGTGTTG -3’ |  |  |
|  |  |  |  |  |
| *CETN2* | Fw: | 5'- catggcatcaagttctcagcga -3' | quantitative real-  time RT-PCR | 58 |
|  | Rv: | 5'- catcacagttaaaaagtcacca -3' |  |  |
|  |  |  |  |  |
| *DDB1* | Fw: | 5'- TTGCACTTGAGGAATGGTGA -3' | quantitative real-  time RT-PCR | 58 |
|  | Rv: | 5'- GGACCATTTGTTTGTCGTTC -3' |  |  |
|  |  |  |  |  |
| *ERCC1* | Fw: | 5'- CAATCCCGTACTGAAGTTCGT -3' | quantitative real-  time RT-PCR | 58 |
|  | Rv: | 5'- TGGGTGCAGGTTGTGGTAG -3' |  |  |
|  |  |  |  |  |
| *ERCC2* | Fw: | 5'- CCAAAGGCTTCACCATCATCA -3' | quantitative real-  time RT-PCR | 59 |
|  | Rv: | 5'- ACAGACTGGAAACGCTCAAAATA -3' |  |  |
|  |  |  |  |  |
| *ERCC4* | Fw: | 5'- CTGGAACACTGAGAAGAAACATC -3' | quantitative real-  time RT-PCR | 64 |
|  | Rv: | 5'- GCAGACAGGCAGAAGTATGG -3' |  |  |
|  |  |  |  |  |
| *ERCC8* | Fw: | 5'- AGCAAACACTGCTCATAATGGG -3' | quantitative real-  time RT-PCR | 62 |
|  | Rv: | 5'- GAACTATTCCAGAGCCTCATTCG -3' |  |  |
|  |  |  |  |  |
| *ERCC5* | Fw: | 5'- AAGTGTGTGCTGGGGGATGA -3' | quantitative real-  time RT-PCR | 60 |
|  | Rv: | 5'- CAGTAAACGGTATTCCTTTTCCAT -3' |  |  |
|  |  |  |  |  |
| *XPA* | Fw: | 5'- GGGGTGATATGAAACTCTACTTAAAG -3' | quantitative real-  time RT-PCR | 60 |
|  | Rv: | 5'- CCTGTCGGACTTCCTTTGC -3' |  |  |
|  |  |  |  |  |
| *GAPDH* | Fw: | 5'- AGCCACATCGCTCAGACAC -3' | quantitative real-  time RT-PCR | 60 |
|  | Rv: | 5'- GCCCAATACGACCAAATCC -3' |  |  |
|  |  |  |  |  |
| *PBGD* | Fw: | 5'- TGCAACGGCGGAAGAAAACA -3' | quantitative real-  time RT-PCR | 60 |
|  | Rv: | 5'- GCAGATGGCTCCGATGGTG -3' |  |  |
|  |  |  |  |  |
| *USP7* | Fw: | 5'- CGGCCTGATGCTTTTTGGAC -3' | quantitative real-  time PCR | 53 |
|  | Rv: | 5′- TGAGAGCCGGTACATCAGGA -3’ |  |  |
|  |  |  |  |  |
| *HMGN1* | Fw: | 5'- AGAGACGGAAAACCAGAGTCC -3' | quantitative real-  time PCR | 64 |
|  | Rv: | 5′- CGTGATGGATGCTTAGTCGG-3’ |  |  |
|  |  |  |  |  |
| *XAB2* | Fw: | 5'- AACGAGCTGGGACCCTCAG -3' | quantitative real-  time PCR | 62 |
|  | Rv: | 5′- TATCAGTTTTTGGGGGCCGAGT -3’ |  |  |
|  |  |  |  |  |
| *MUTY* | Fw: | 5'- ATGACACCGCTCGTCTCC -3' | quantitative real-  time PCR | 60 |
|  | Rv: | 5′- GCTTCTGCCTCCCTTCCT -3’ |  |  |
|  |  |  |  |  |
| *XRCC1* | Fw: | 5’- GGGACCGGGTCAAAATTGTT -3’ | quantitative real-  time PCR | 62 |
|  | Rv: | 5’- ACCGTACAAAACTCAAGCCAAAG -3’ |  |  |
|  |  |  |  |  |
| *OGG1* | Fw: | 5’- GTGCCCGTTACGTGAGTGCCAGTGC -3’ | quantitative real-  time PCR | 63 |
|  | Rv: | 5’-AGAGAAGTGGGGAATGGAGGGGAAGGTG T -3’ |  |  |
|  |  |  |  |  |
| *MPG* | Fw: | 5’- TTTACGGCATGTACTTCTGCAT -3’ | quantitative real-  time PCR | 62 |
|  | Rv: | 5’- ATGGTCTCCAGACCTTCCAG -3’ |  |  |
|  |  |  |  |  |
| *TDG* | Fw: | 5’- TGAAGCTCCTAATATGGCAGTT -3’ | quantitative real-  time PCR | 60 |
|  | Rv: | 5’- TTCCACTGGTTGTTTTGGTT -3’ |  |  |
|  |  |  |  |  |
| *NEIL2* | Fw: | 5’- GGGGCAGCAGTAAGAAGCTA -3’ | quantitative real-  time PCR | 64 |
|  | Rv: | 5’- GGAATAATTTCTTTCCATGGACCT -3’ |  |  |
|  |  |  |  |  |
| *NEIL3* | Fw: | 5’- GCAGAATAACTGTGTGCCGCT -3’ | quantitative real-  time PCR | 66 |
|  | Rv: | 5’- ACCCTGCTAGATGTCCAACTGATT -3’ |  |  |
|  |  |  |  |  |
| *CCNH* | Fw: | 5’- GCATTGACGGATGCTTACCT -3’ | quantitative real-  time PCR | 60 |
|  | Rv: | 5’- TGACATCGCTCCAACTTCTG -3’ |  |  |
|  |  |  |  |  |
| *MNAT1* | Fw: | 5’- TGGCCGACAAGCACGCAACC -3’ | quantitative real-  time PCR | 64 |
|  | Rv: | 5’- GGAGCGGGAGGATTTCTTCT -3’ |  |  |
|  |  |  |  |  |
| *XAB2* | Fw: | 5’- CCCCCAAAATATGCCAAGACCT -3’ | quantitative real-  time PCR | 58 |
|  | Rv: | 5’- TGCTCGTCCGACAGCACCTC -3’ |  |  |
|  |  |  |  |  |
| *RAD52* | Fw: | 5’- CACAGACACGACGTCCATTG -3’ | ChIP (E2F1) | 60 |
|  | Rv: | 5’- ACAAAGCGCTTTGTCCCAAG -3’ |  |  |
|  |  |  |  |  |
| *RAD52* | Fw: | 5’- GGTCGCATTACGCTTAGCTC -3’ | ChIP (NC) | 60 |
|  | Rv: | 5’- ATCCACCCAAAGCAGTCAACV\ -3’ |  |  |

***Abbreviations***: Fw: Forward; Rv: Reverse,

RT-PCR: Reverse Transcription-Polymerase Chain Reaction;

ChIP: Chromatin Immunoprecipitation

NC: Negative Control; Ann. Temp: Annealing Temperature.

**Suppl Table 4. List of siRNAs and vectors employed**

| ***siRNAs*** |  | *Reference* | *cat. #* |
| --- | --- | --- | --- |
|  |  | HSS103015 |  |
|  | si-E2F1 | HSS103016 | 1299001 |
|  |  | HSS103017 | (Thermo Scientific) |
|  |  |  |  |
|  |  | HSS109021 |  |
|  | si-RAD52 | HSS109023 | 1299001 |
|  |  | HSS184195 | (Thermo Scientific) |
| ***Plasmids*** |  | *Reference* | *Supplied by* |
|  | HA-IsceI | **-** | Soutoglou E. |
|  | YFP-RAD52 | Ochs et al., 2016 | Lucas J. |
|  | DR-GFP | Stark J. et al. 2004 | Halazonetis T. |
|  | BIR-GFP | Sotiriou et al., 2014 | Halazonetis T. |
|  | SA-GFP | Stark J. et al. 2004 | Halazonetis T. |
|  | GFP-POLK | **-** | Lygerou Z. |
